# MediMer: a versatile do-it-yourself peptide-receptive MHC class I multimer platform for tumor neoantigen-specific T cell detection

**DOI:** 10.3389/fimmu.2023.1294565

**Published:** 2024-01-04

**Authors:** Marten Meyer, Christina Parpoulas, Titouan Barthélémy, Jonas P. Becker, Pornpimol Charoentong, Yanhong Lyu, Selina Börsig, Nadja Bulbuc, Claudia Tessmer, Lisa Weinacht, David Ibberson, Patrick Schmidt, Rüdiger Pipkorn, Stefan B. Eichmüller, Peter Steinberger, Katharina Lindner, Isabel Poschke, Michael Platten, Stefan Fröhling, Angelika B. Riemer, Jessica C. Hassel, Maria Paula Roberti, Dirk Jäger, Inka Zörnig, Frank Momburg

**Affiliations:** ^1^ Antigen Presentation and T/NK Cell Activation Group, German Cancer Research Center (DKFZ), Heidelberg, Germany; ^2^ Clinical Cooperation Unit Applied Tumor Immunity, DKFZ, Heidelberg, Germany; ^3^ Department of Medical Oncology, National Center for Tumor Diseases (NCT) Heidelberg, Heidelberg University Hospital, Heidelberg, Germany; ^4^ Division of Immunotherapy and Immunoprevention, DKFZ, Heidelberg, Germany; ^5^ German Center for Infection Research (DZIF) Partner Site Heidelberg, Heidelberg, Germany; ^6^ Center for Quantitative Analysis of Molecular and Cellular Biosystems (Bioquant), Heidelberg University, Heidelberg, Germany; ^7^ Deep Sequencing Core Facility, Heidelberg University, Heidelberg, Germany; ^8^ GMP and T Cell Therapy, DKFZ, Heidelberg, Germany; ^9^ Division of Immune Receptors and T Cell Activation, Center for Pathophysiology, Infectiology, Medical University of Vienna, Vienna, Austria; ^10^ Clinical Cooperation Unit Neuroimmunology and Brain Tumor Immunology, DKFZ, Heidelberg, Germany; ^11^ Immune Monitoring Unit, NCT Heidelberg and DKFZ, Heidelberg, Germany; ^12^ German Cancer Consortium (DKTK), DKFZ, Core Center, Heidelberg, Germany; ^13^ Department of Neurology, Medical Faculty Mannheim, Mannheim Center for Translational Neuroscience (MCTN), Heidelberg University, Mannheim, Germany; ^14^ DKFZ Hector Cancer Institute at the University Medical Center, Mannheim, Germany; ^15^ Helmholtz Institute for Translational Oncology, Mainz (HI-TRON Mainz), Mainz, Germany; ^16^ Division of Translational Medical Oncology, NCT Heidelberg and DKFZ, Heidelberg, Germany; ^17^ Institute of Human Genetics, Heidelberg University, Heidelberg, Germany; ^18^ Section of DermatoOncology, Department of Dermatology and NCT, Heidelberg University Hospital, Heidelberg, Germany

**Keywords:** T cells, tumor immunotherapy, peptide-MHC class I multimer, neoepitope screening, T cell receptor discovery, tumor neoantigen, personalized medicine

## Abstract

Peptide-loaded MHC class I (pMHC-I) multimers have revolutionized our capabilities to monitor disease-associated T cell responses with high sensitivity and specificity. To improve the discovery of T cell receptors (TCR) targeting neoantigens of individual tumor patients with recombinant MHC molecules, we developed a peptide-loadable MHC class I platform termed MediMer. MediMers are based on soluble disulfide-stabilized β_2_-microglobulin/heavy chain ectodomain single-chain dimers (dsSCD) that can be easily produced in large quantities in eukaryotic cells and tailored to individual patients’ HLA allotypes with only little hands-on time. Upon transient expression in CHO-S cells together with ER-targeted BirA biotin ligase, biotinylated dsSCD are purified from the cell supernatant and are ready to use. We show that CHO-produced dsSCD are free of endogenous peptide ligands. Empty dsSCD from more than 30 different HLA-A,B,C allotypes, that were produced and validated so far, can be loaded with synthetic peptides matching the known binding criteria of the respective allotypes, and stored at low temperature without loss of binding activity. We demonstrate the usability of peptide-loaded dsSCD multimers for the detection of human antigen-specific T cells with comparable sensitivities as multimers generated with peptide-tethered β_2_m-HLA heavy chain single-chain trimers (SCT) and wild-type peptide-MHC-I complexes prior formed in small-scale refolding reactions. Using allotype-specific, fluorophore-labeled competitor peptides, we present a novel dsSCD-based peptide binding assay capable of interrogating large libraries of *in silico* predicted neoepitope peptides by flow cytometry in a high-throughput and rapid format. We discovered rare T cell populations with specificity for tumor neoepitopes and epitopes from shared tumor-associated antigens in peripheral blood of a melanoma patient including a so far unreported HLA-C*08:02-restricted NY-ESO-1-specific CD8^+^ T cell population. Two representative TCR of this T cell population, which could be of potential value for a broader spectrum of patients, were identified by dsSCD-guided single-cell sequencing and were validated by cognate pMHC-I multimer staining and functional responses to autologous peptide-pulsed antigen presenting cells. By deploying the technically accessible dsSCD MHC-I MediMer platform, we hope to significantly improve success rates for the discovery of personalized neoepitope-specific TCR in the future by being able to also cover rare HLA allotypes.

## 1 Introduction

Detection of antigen-specific T cells labeled by recombinantly produced soluble peptide-loaded MHC-I (pMHC-I) molecules using flow cytometry represents to date one of the most sensitive techniques for identifying, monitoring and quantifying T cell responses against well-defined antigens. The pioneering work by Altman and colleagues demonstrated that insufficient binding affinities and high off-rates of soluble monomeric pMHC-I complexes for T cell receptor labeling can be overcome by streptavidin-mediated multimerization of biotinylated MHC-I heavy ectodomains assembled with β_2_-microglobulin (β_2_m) and an appropriate peptide ligand (1). Soluble pMHC-I multimer staining reagents have since undergone an evolutionary process regarding their production process, multimer valency and methods of peptide loading as well as strategies that allow multiplex detection of various T cell specificities within one labeling reaction by combinatorial color encoding and DNA barcoding of multimers (2–6).

Interrogation of large numbers of potential shared or individual T cell antigens across the human population with pMHC-I multimer reagents however remains a challenging issue and is often limited by the capacity to manufacture individualized pMHC-I multimer libraries and to cover a broad spectrum of HLA allotypes, which is addressed by virtue of a pMHC-I platform.

Originally, bacterially expressed soluble MHC-I heavy chains and β_2_m were combined in rather inefficient *in vitro* pMHC-I folding reactions in the presence of a chemically synthesized peptide ligand leading to the necessity of size exclusion chromatography for each individual pMHC-I followed by enzymatic BirA biotin ligase-mediated biotinylation and multimer formation with fluorochrome-conjugated streptavidin (1). This widely used method has been optimized and miniaturized by firstly purifying correctly oxidized heavy chains of *in vivo* biotinylated MHC-I ectodomains isolated from *E. coli* inclusion bodies leading to higher *in vitro* folding efficiencies when combined with synthetic peptides and β_2_m in small-scale refolding reactions, which can be directly multimerized without further purification steps (7), and has recently been used for the identification of immunogenic glioblastoma-specific T cell epitopes derived from transposable elements (8).

By introducing an additional disulfide bond locking the C-terminal end of the peptide binding cleft in MHC-I molecules, Springer and coworkers achieved peptide-independent stabilization of the MHC-I heavy chain (9). Disulfide-stabilized A*02:01 heavy chains molecules showed an unaltered structure of the peptide-binding groove in the peptide-bound and peptide-free states as well as unaffected recognition by an A*02:01-restricted TCR (10–12). Here, *E. coli*-derived disulfide-stabilized MHC-I heavy chains were refolded in the presence of a dipeptide and β_2_m to subsequently purify soluble peptide-free MHC-I molecules that are highly peptide-receptive (10). Alternatively, various peptide exchange-based pMHC-I platforms have been proposed, including temperature-induced exchange (13) and a widely used peptide exchange system, utilizing the *in vitro* refolding of MHC-I with β_2_m and a conditional placeholder peptide ligand, that are cleaved upon exposure to ultraviolet light (14). Decomposed placeholder peptides rapidly dissociate from the peptide binding groove, thus allowing any other suitable peptide ligand of interest to rescue the heterotrimeric complex. This platform has been successfully applied for the generation of larger pMHC-I libraries (15–17) but is yet commercially available for only a very limited number of HLA alleles.

A different strategy pioneered by Hansen and colleagues makes use of fusion proteins that genetically encode the entire heterotrimeric peptide–β_2_m–heavy chain complex linking the components through flexible glycine-serine sequences (18). Such MHC-I single-chain trimers (SCT) have been optimized for peptide binding stability and linker accommodation by introduction of a disulfide trap between a cysteine-substituted conserved tyrosine residue at the C-terminal end of the MHC-I α1 helix with another cysteine in the peptide–β_2_m linker (19–21). Disulfide-trapped SCT (dtSCT) feature functionally correct folding, improved thermal stability and complete exclusion of competitor peptides when produced as soluble molecules in bacteria or expressed in the natural membrane-bound form in cell lines and perform excellently in pMHC-I multimers (19, 22). Dimeric SCT tethered to the heavy chain of IgG were successfully used to detect antigen-specific T cells (23, 24). We have previously reported soluble dimeric dtSCT fused to the Fc portion of IgG that are efficiently producible in suspension-adapted Chinese Hamster Ovary (CHO-S) cells (25). While stable peptide binding in SCT is advantageous for their use as vaccines (26–28), the generation of larger SCT libraries for the screening of pMHC-I reactive T cells is technically demanding since each SCT needs to be genetically engineered separately (29, 30).

In screening campaigns for tumor neoepitope-reactive T cells with peptide-loaded MHC multimers we encountered the need to cover a variety of rare HLA-A, -B, and -C allotypes that are presently not commercially available as recombinant MHC-I molecules. In order to fully exploit the tumor patients’ T cell repertoire directed against all six HLA-A,B,C allotypes, we developed a new platform termed MediMer (MHC-I, empty, single-chain dimer) based on disulfide-stabilized peptide-free β_2_m–MHC-I heavy chain single-chain dimers (dsSCD) that are produced in metabolically biotinylated form by CHO-S producer cells without the need of additional steps such as *in vitro* refolding. The MediMer production system can be easily adapted and allows fast do-it-yourself tailoring of required HLA allotypes for a given patient cohort. Purified dsSCD representing a so far unrestricted number of HLA-A,B,C allotypes, are highly stable and peptide-receptive, making them highly suitable for the screening of individualized neoepitope large peptide libraries. Furthermore, we show that multimerized peptide-loaded dsSCD perform efficiently to label and isolate antigen-specific T cells and can be combined in a single experiment in a complementary manner with other commercial pMHC-I platforms and multiplex labeling strategies such as DNA-barcoding for single cell RNA sequencing. In addition, we present a dsSCD-based peptide binding assay for the fast high-throughput binding validation of *in silico* predicted HLA ligands.

## 2 Materials and methods

### 2.1 Cloning of dsSCD and dtSCT expression plasmids

Disulfide-stabilized human β_2_-microglobulin (β_2_m)-HLA-A,B,C single-chain dimers were cloned by assembling:

1. the modified influenza A virus hemagglutinin H1N1 leader sequence (MAKANLLVLLCALAAADALGS),
2. the leader-less human β_2_-microglobulin sequence (accession no. AK315776, Ile_21_-Met_119_),
3. a glycine-serine linker sequence (SGGS[GGGGS]_3_ASGGG),
4. leader-less ectodomains of various HLA-A,B,C alleles (**Supplementary Table 4**), Gly_1_-Pro_283_ containing Tyr_84_ to Cys and Ala_139_ to Cys mutations), forming an additional disulfide bond between α1 and α2 domains (9, 31),
5. the tag-linker sequence including a His_8_-tag (**bold**, *italics*), a BirA biotin ligase recognition site (**bold**) and a double thrombin protease cleavage site (*italics*) (32) (TSTGQL ***HHHHHHHH*** QL**GLNDIFEAQKIEWHE** *LVPRSLVPRS*TS),
6. the Fc portion of mouse IgG2a (BC031479; Glu_215_-Lys_447_, Cys_224_/Ser), and
7. a *C*-terminal StrepTag-II (**bold**) with adapter sequences (DPG**WSHPQFEK**SR) by restriction enzyme cloning and PCR mutagenesis. In Fc-free dsSCD constructs the tag-linker sequence was terminated with 2 stop codons after the BirA biotin ligase recognition site. Assembled cDNA sequence were cloned between the *Nhe*I and *Xba*I sites of expression vector pcDNA3.1(+) (Invitrogen).

Disulfide-trapped single-chain trimer (dtSCT) constructs were cloned by assembling:

- the leader sequence MAKANLLVLLCALAAAQPAMA,
- a sequence encoding an 8-11-mer peptide of choice,
- glycine-serine linker #1 containing a cysteine residue at position 2 (G**C**GSGGGGAPGSGGGS),
- the leader-less β_2_m sequence,
- glycine-serine linker #2 (SGGS[GGGGS]_3_ASGGG),
- leader-less ectodomains of various HLA-A,B,C alleles containing a Tyr_84_ to Cys mutation to form the disulfide trap with Cys_2_ in linker #1 (19) followed by the tag-linker and mIgG2a-Fc as described above and as previously described for dtSCT in (25, 32).

In the HLA-A*02:01 dsSCD with cleavable β_2_m linker, the human rhinovirus (HRV) type 14 3C protease cleavage site (bold) was inserted in a modified glycine-serine linker (SGGS[GGGGS]_3_AS**LEVLFQGP**SGAS). *E. coli* BirA biotin ligase (accession no. M15820) was cloned by PCR from genomic DNA isolated *E. coli* XL1-Blue and the coding sequence except for Met_1_ was cloned at the 3’ end of an Igk leader sequence in the expression vector pcDNA3.1 (–). The KDEL motif coding for an ER retention/recycling signal was cloned at the C-terminal end of the open reading frame after Glu_270_.

### 2.2 Soluble dsSCD and dtSCT synthesis in mammalian cell transient gene expression systems

Expression of dsSCD and dtSCT was tested in suspension-adapted FreeStyle™ Chinese hamster ovary cell (CHO-S, Gibco) as previously described (25, 33) and FreeStyle™ 293-F (293-F, Gibco) transient gene expression systems (32). CHO-S were routinely cultured in PowerCHO-2 CD (Lonza), supplemented with 8 mM GlutaMAX™ (Gibco) and 0.5x Antibiotic Antimycotic solution (Anti-Anti, Sigma-Aldrich) at 37°C, 8% CO_2_ and 100 rpm with a 50 mm shaking diameter. 293-F cells were routinely cultured in FreeStyle™ 293 Expression Medium (293-F medium, Gibco) at 37°C, 8% CO_2_ and 100 rpm. For protein production in CHO-S, CHO-S cells were resuspended at 3x10^6^ cells/ml in ProCHO4 medium (Lonza) supplemented with 4 mM GlutaMAX™ (Gibco), 4 µg/ml D-biotin (Sigma-Aldrich) and 0.5x Anti-Anti followed by the sequential addition per 1x10^6^ cells of 2.5 μg 25-kDa linear polyethyleneimine (PEI; Polysciences), 0.32 μg plasmid DNA encoding for a dsSCD or dtSCT and 0.32 μg plasmid DNA encoding for an ER-retained BirA ligase (BirA_KDEL_). Co-transfected CHO-S were maintained at 37°C, 8% CO_2_ and 100 rpm for 6 hours followed by supplementation with 1 mM valproic acid (VPA, Sigma-Aldrich) and maintenance for 6 days under hypothermic conditions at 32°C, 5% CO2 and 100 rpm. For protein production in 293-F cells, 293-F cells were resuspended at 1x10^6^ cells/ml in 293-F medium supplemented with 4 mM GlutaMAX™, 4 µg/ml D-biotin following co-transfection with dsSCD or dtSCT encoding plasmids and BirA_KDEL_ plasmid using the 293-free transfection reagent (Sigma-Aldrich) according to the manufacturer’s protocols. The next day, VPA was added to the 293-F transfected culture to a final concentration of 4 mM as well as 0.5x anti-anti. The supplemented 293-F culture was further maintained for 6 days at 37°C, 8% CO_2_ and 100 rpm. On day 6 post transfection, cell-free CHO-S or 293-F supernatants of dsSCD-Fc or dtSCT-Fc transfections were supplemented with 0.1 volumes of 10x Dulbecco’s PBS (DPBS) (Sigma-Aldrich) and 2 IU thrombin (Merck) per mg dsSCD-Fc or dtSCT-Fc, quantified by a mouse-IgG-Fc-based sandwich ELISA, followed by an overnight incubation at 37°C. Soluble monomeric Fc-free dtSCT from 293-F cultures or dsSCD from CHO-S cultures were further purified by immobilized metal affinity chromatography (IMAC) using Ni-INDIGO MagBeads (Cube Biotech) or column-packed Ni Sepharose Excel resin (Cytiva), respectively, according to the manufacturers’ instructions. Eluted proteins were finally dialyzed against PBS (pH 7.4) and their purity and metabolic biotinylation were verified by a non-reducing 10% SDS-PAGE (Invitrogen) in the presence of streptavidin. Purified dsSCT and dsSCD were stored in PBS at 4°C throughout the study unless otherwise stated. In one experiment purified dsSCD was supplemented with 5% glycerol (v/v) and 0.5% bovine serum albumin (BSA) (w/v) in PBS and stored at -20°C prior to its usage for cell stainings. For expression analysis of CHO-S and 293-F cells by intracellular staining, an aliquot of cells was taken 48 h post transfection and washed once with DPBS followed by labeling with the Zombie Aqua™ Fixable Viability Kit (BioLegend, 1:300) to exclude dead cells. Cells were fixed and permeabilized for 10 min at 4°C and were then stained intracellularly for 30 min with anti-HLA-A2-APC (BB7.2, BioLegend, 343308) using the Cytofix/Cytoperm™ Fixation/Permeabilization Kit (BD Bioscience) according to the manufacturer’s instructions. Stained cells were washed once with DPBS + 2% fetal calf serum (FCS) and acquired on a FACSCanto™ II flow cytometer (BD Bioscience). Analysis was done using the FlowJo Software (BD Bioscience). Single living (ZombieAqua^–^) cells were gated and anti-HLA-A2-APC signals visualized.

### 2.3 Peptide synthesis

For the fast and reliable peptide binding validation of freshly produced dsSCD of various HLA-A, B, C allotypes, a set of peptide sequences of known HLA-I ligands (**Supplementary Table 1**) were rationally modified to incorporate a fluorescein-5-isothiocyanate (FITC)-conjugated lysine residue (K^FITC^/K*) at a selected non-anchor residue.

Chemical peptide synthesis was performed employing the Fmoc strategy (34, 35) in a fully automated multiple synthesizer Syro II (MultiSyn Tech, Germany). The synthesis was carried out on preloaded 2-CT-polystyrene resin (Rapp Polymere GmbH, Germany). As coupling agent 2-(1H-benzotriazole-1-yl)-1,1,3,3-tetramethyluronium hexafluorophosphate (HBTU) was used. For the FITC-conjugated peptides we used Fmoc-Lys(5-FITC)-OH (Biomol GmbH, Germany). The synthesized peptides were cleaved and deprotected from the solid support by treatment with 90% trifluoroacetic acid, 8% tri-isopropylsilane, and 2% water (v/v/v) for 2.5 h at room temperature. The products were precipitated in ether and checked by analytical LC/MS (Thermo Finnigan LCQ). When necessary, peptides were purified by preparative HPLC on a Kromasil 100–10C 10 µm 120A reverse phase column (20 x 150 mm) using an eluent of 0.1% trifluoroacetic acid in water (A) and 80% acetonitrile in water (B). The peptide was eluted with a successive linear gradient of 10% B to 80% B in 30 min at a flow rate of 17 ml/min. The fractions corresponding to the purified peptide were lyophilized. The purified material was characterized by analytical LC/MS (Thermo Finnigan LCQ).

K^FITC^-containing peptides as well as selected unlabeled peptides were dissolved to 10 mM and 50 mM in dimethyl sulfoxide (DMSO), respectively. In addition, peptides employed for immunogenicity screening of predicted neoepitopes of the melanoma patient were purchased from JPT Peptide Technologies GmbH (Berlin, Germany).

### 2.4 DsSCD peptide-binding assays

To verify binding of selected ^FITC^peptides to dsSCD, dsSCD were diluted to 33 nM in 30 µl DPBS supplemented with 5% glycerol (v/v) and 0.5% BSA (w/v) and 1 µl streptavidin-conjugated beads (Spherotech, SVP-60-05) followed by the addition of 1 µM ^FITC^peptide. ^FITC^peptide-pulsed dsSCD-loaded beads were incubated overnight in the dark at room temperature (RT) under shaking conditions in V-bottom 96-well plates. After the incubation, beads were washed twice with DPBS + 0.5% BSA (w/v) + 0.1% Tween-20 (v/v) and once with DPBS + 2% FCS prior their acquisition on a LSRFortessa™ flow cytometer (BD Bioscience). In some experiments, the ^FITC^peptide concentration or pulse duration or incubation temperature was varied as indicated in the figure legends. To assess the binding of a given unlabeled test peptide to a dsSCD, the selected dsSCD was immobilized at 33 nM on beads as described above and pulsed overnight (ca. 18 h) with 10 µM of the test peptide or were left empty serving as maximum ^FITC^peptide loading control (“median fluorescence intensity (MFI) Max”) in the next step. After the overnight incubation, 1 µM of an established dsSCD binding ^FITC^peptide was added for 10 min as a competitor directly to the dsSCD-beads in the presence of 10 µM test peptide as well as to dsSCD-beads lacking a prior peptide pulse. dsSCD-beads were washed immediately after the 10min ^FITC^peptide pulse and subjected to flow cytometric analysis. Unlabeled test peptide occupancy of dsSCD-beads was assessed based on FITC MFI values after ^FITC^peptide pulse (“MFI Test”) relative to dsSCD-beads that had been loaded with ^FITC^peptide in the absence of a test peptide. For normalization, background MFI of empty beads was subtracted. % MFI reduction was calculated according the following equation:


$$\text{\% }MFI\;reduction=\frac{\left(Normalized\;MFI\;Max-Normalized\;MFI\;Test\right)}{\left(Normalized\;MFI\;Max\right)}\;x\;100$$

### 2.5 EasYmer peptide-HLA-I complex formation assay

HLA-A*01:01, HLA-A*02:01, HLA-A*68:01, HLA-B*08:01, HLA-B*14:01, HLA-C*05:01 and HLA-C*07:01 easYmer^®^ kits were purchased from immunAware ApS (Horsholm, Denmark). Peptide interaction with a given easYmer composed of soluble biotinylated MHC-I heavy chain and non-covalently associated β_2_m (7) was detected by a flow cytometry-based peptide-HLA-I complex formation assay according to the manufacturer’s protocol. Briefly, easYmers were diluted to 0.5 µM in provided folding buffer and a library of peptides including a designated positive-binding control peptide per HLA allotype was added at a final concentration of 3 µM, or diluted easYmers were left in the absence of peptide as negative control for 2-3 days at RT. The folding reaction was diluted to 5 nM in a final volume of 60 µl DPBS + 5% glycerol and supplemented with streptavidin-conjugated beads (Spherotech, SVP-60-05) (finally diluted 1:135), following an incubation for 1 h at 37°C under constant shaking. EasYmer-loaded beads were washed three times with FACS Buffer (DPBS + 2% FCS) and were stained with anti-β2m-PE (clone BBM.1, Santa Cruz sc-13565 PE) for 30 min at 4°C. Beads were again washed three times with FACS buffer and acquired on a LSRFortessa (BD Bioscience) flow cytometer. Successful peptide-HLA-I complex formation was assessed as an approximation by using the bead-immobilized median fluorescence intensity (MFI) of β2m after addition of a positive control peptide relative to the MFI value of a given test peptide according to the following equation:


$$\text{\% }easYmer\;formation=\frac{MFI\;value\;of\;easYmer\;folding\;reaction\;with\;test\;peptide}{MFI\;value\;of\;easYmer\;folding\;reaction\;with\;positive\;ctrl.\;peptide}\;x\;100$$

### 2.6 Data-independent acquisition mass spectrometry of empty and peptide-loaded dsSCD

CHO cell-derived HLA-A*02:01 dsSCD diluted to 0.18 µg/µL (3 µM) in DPBS was loaded overnight at room temperature with 100 µM of a peptide pool comprising the known HLA-A*02:01 binder NLVPMVATV, VLEETSVML, GLCTLVAML, GILGFVFTL, YLQPRTFLL, ELAGIGILTV and HLA-A*02:01 non-binder CTELKLSDY (**Supplementary Table 3**) or the diluted HLA-A*02:01 dsSCD was left overnight without external peptide addition. HLA-A*02:01 dsSCD samples were mixed with 1 ml lysis buffer (0.25% sodium deoxycholate, 1% N-octyl-β-D-glucopyranoside, 1 mM PMSF, 1 mM EDTA, 0.2 mM iodoacetamide, 1 cOmplete™ Protease Inhibitor Cocktail Mini tablet (Roche) per 5 ml of lysis buffer) and directly used for immunoprecipitation with an HLA-A2-specific antibody (clone BB7.2) as previously described with minor modifications (36). Lyophilized peptides were dissolved in 12 µl of 5% acetonitrile (ACN) in 0.1% trifluoroacetic acid (TFA) and spiked with 100 fmol Peptide Retention Time Calibration (PRTC) Mixture (Pierce) and transferred to QuanRecovery Vials with MaxPeak HPS (Waters, Milford, MA, USA). All samples were analyzed using an UltiMate 3000 RSLCnano system coupled to an Orbitrap Exploris 480 equipped with a FAIMS Pro Interface (Thermo Fisher Scientific). For chromatographic separation, peptides were first loaded onto a trapping cartridge (Acclaim PepMap 100 C18 μ-Precolumn, 5 μm, 300 μm i.d. x 5 mm, 100 Å; Thermo Fisher Scientific) and then eluted and separated using a nanoEase M/Z Peptide BEH C18 130A 1.7µm, 75µm x 200mm (Waters). Total analysis time was 120 min and separation was performed using a flow rate of 0.3 µl/min with a gradient starting from 1% solvent B (100% ACN, 0.1% TFA) and 99% solvent A (0.1% formic acid (FA) in H_2_O) for 0.5 min. Concentration of solvent B was increased to 2.5% in 12.5 min, to 28.6% in 87 min and then to 38.7% in 1.4 min. Subsequently, concentration of solvent B was increased to 80% in 2.6 min and kept at 80% solvent B for 5 min for washing. Finally, the column was re-equilibrated at 1% solvent B for 11 min. The LC system was coupled on-line to the mass spectrometer using a Nanospray-Flex ion source (Thermo Fisher Scientific), a SimpleLink Uno liquid junction (FossilIonTech) and a CoAnn ESI Emitter (Fused Silica 20 µm ID, 365 µm OD with orifice ID 10 µm; CoAnn Technologies). The mass spectrometer was operated in positive mode and a spray voltage of 2500 V was applied for ionization with an ion transfer tube temperature of 300°C. For ion mobility separation, the FAIMS module was operated with standard resolution and a total carrier gas flow of 4.6 l/min. Each sample was injected twice using either a compensation voltage of -50 V or -70 V for maximal orthogonality and thus increased immunopeptidome coverage. Full Scan MS spectra were acquired for a range of 300 – 1650 m/z with a resolution of 120.000 (RF Lens 50%, AGC Target 300%). MS/MS spectra were acquired in data-independent mode for a cycle time of 3s using 44 previously determined dynamic mass windows optimized for HLA class I peptides with an overlap of 0.5 m/z. HCD collision energy was set to 28% and MS/MS spectra were recorded with a resolution of 30.000 (normalized AGC target 3000%).

MS raw data was analyzed using the directDIA workflow of the Spectronaut software [version 17; Biognosys (37)] and searched against the UniProtKB/TrEMBL database for *Cricetulus griseus* (Chinese hamster) (retrieved: 12.09.2022, 78117 entries) and a database containing the seven peptides used for external loading of the dsSCD. Search parameters were set to non-specific digestion and a peptide length of 7-15 amino acids. Carbamidomethylation of cysteine and oxidation of methionine were included as variable modifications. Additionally, MS raw data were manually searched using Skyline (version 22) (38). Spectral libraries for peptides originating from the peptide pool used for loading were *in silico* generated using PROSIT (39). Spectral angles were calculated as described previously (40). All results were visualized using in-house developed R scripts.

### 2.7 TCR cloning and generation of stably recombinant TCR expressing CD8^+^ Jurkat 76 T cell lines

Sequences of published T cell receptors (TCR) were cloned as chimeric human (h) TRVB–mouse (m) TRBC and hTRAV–mTRAC sequences in bicistronic open reading frames containing a P2A or T2A ribosomal skipping sequence between TCRβ and TCRα chains. The HCMV pp65_495-503_/HLA-A*02:01 specific TCR RA14 (41), the MART1_27-35_/HLA-A*02:01 specific TCR DMF5 (42), the KRAS^G12V^_8-16_/HLA-A*11:01 specific TCR Ry-4148-G12V-9mer (43), the KRAS^G12D^_10-18_/HLA-C*08:02 specific TCR TCR4(G12D) (44), the NY-ESO-1_157-165_/HLA-A*02:01 specific TCR 1G4 (45), and the NY-ESO-1_96-104_/HLA-C*03:04 specific TCR 3C7 (46) have been described previously. For this study, 5 additional TCRs following the cloning strategy mentioned above (MM-01 – MM-05) were cloned using sequence information from the vloupe.vloupe output file of a 10x Genomics-based single-cell sequencing data set of pMHC-I multimer^+^ CD8^+^ T cell populations that have been cell sorted from peripheral blood of a melanoma patient. All TCR sequences were subcloned in the transposon expression vector pSBbi-pur (47) (addgene plasmid #60523) together with pcDNA3.1(+) expression vector encoding SB100 Sleeping Beauty transposase (48) subcloned from pCMV(CAT)T7-SB100 (addgene plasmid #34879). To test the recombinant TCRs for pMHC-I multimer binding, a J76 Jurkat E6.1 subline expressing CD8 and lacking endogenous TCRα and β chains [J76^CD8+^, generated as described (49)] was electroporated with 2 µg endotoxin-free TCR plasmid and 2 µg transposase plasmid per 2x10^6^ J76^CD8+^ cells using the P3 primary cell 4D-Nucleofector X Kit S (Lonza) according to the manufacturer’s recommended protocol. TCR and CD8 expression was monitored by flow cytometry using antibodies against murine TCR-Cβ-APC (clone H57-597, BioLegend 109212) and CD8-PacificBlue (Clone SK1, BioLegend 344718). TCR-transfected J76^CD8^ cells were maintained for one week in RPMI supplemented with 10% FCS, 10 µg/ml gentamycin and 2 mM GlutaMAX at 37°C, 5% CO_2_, followed by enrichment for TCR-expressing J76^CD8^ cells using CD3-MicroBead-based (Miltenyi) magnetic cell sorting (MACS) according to the manufacture’s protocol prior to their usage in pMHC-I multimer stainings.

### 2.8 Functional validation of TAA-specific TCRs using autologous expanded B cells

Primary human B cells were magnetically isolated from peripheral blood of a melanoma patient using CD19-MicroBeads (Miltenyi). Isolated B cells expanded over the course of 6 days using StemMACS™ HSC medium supplemented with 0.5 µg/ml multimerized soluble human CD40L, 50 IU/ml IL-4, 10 IU/ml IL-2, 10 ng/ml IL-21 (all from Miltenyi), 40 ng/ml BAFF (BioLegend) and 0.625 µg/ml cyclosporin A (Sigma-Aldrich) at 37°C, 5% CO_2_. Expanded B cells were washed three times with DPBS and were co-cultured in a 1:1 ratio with TCR^+^ J76^CD8^ cells in RPMI supplemented with 10% FCS, 10 µg/ml gentamycin and 2 mM GlutaMAX in the presence of cognate and control peptides at various concentrations. After 18 h of co-culture, activation of TCR^+^ J76^CD8^ cells was analyzed by flow cytometry for CD69 upregulation by anti-CD69-PE (FN50, BioLegend 310906) staining combined with anti-murine TCR-Cβ-APC (H57-597) and anti-CD8-Pacific Blue (SK1).

### 2.9 Isolation of primary CD8^+^ T cells from PBMC of healthy donors and a melanoma patient

PBMCs of healthy donors HD-01–HD-07 and an advanced-stage melanoma patient were isolated from blood samples using 1.077 g/ml density gradient centrifugation (Pancoll human, PAN Biotech) and cryopreserved in 90% FCS (Gibco) supplemented with 10% dimethyl sulfoxide (DMSO). Blood samples from healthy donors were collected according to the principles of the Declaration of Helsinki and were obtained from the Deutsches Rotes Kreuz (DRK) Blutspendedienst Baden-Württemberg-Hessen gGmbH, Mannheim, Germany. HLA-A*02:01 expression of HD-01, HD-02 and HD-07 was determined by flow cytometry using an anti-HLA-A2-APC (BB7.2, BioLegend 343308) staining. For HD-03–HD-07 and the melanoma patient a 4-digit NGS-based HLA-typing was performed by the DKMS Life Science Lab gGmbH, Dresden, Germany.

Blood samples from the patient with metastasized melanoma (male, age 47) were obtained by the Section of DermatoOncology (NCT), Heidelberg University Hospital. Tumor lymph node metastases were resected in 08/2021 and subjected to whole exome sequencing and mRNA sequencing in the MASTER program. For all conducted pMHC-I multimer binding analysis of primary human material, PBMCs were thawed and rested overnight in AIM-V™ (Gibco) supplemented with 2% human male AB serum (PAN Biotech) and 1 unit/ml Benzonase® nuclease (Sigma-Aldrich) followed by a magnetic CD8^+^ cell sorting using the REAlease^®^ CD8 MicroBead Kit (Miltenyi) according to the manufacturer’s recommended protocol.

### 2.10 Preparation of single color, dual color-encoded and DNA-barcoded pMHC-I multimer libraries

For multimerization, dsSCD were diluted to 2 µM in DPBS and loaded overnight at RT with indicated peptides at 25 µM. EasYmers^®^ (immunAware) were folded at 2 µM for 2-3 days before multimerization by the addition of 6 µM peptide according to the manufacturer’s protocol. Monomeric dtSCT, folded easYmer at 2 µM and peptide-loaded dsSCD were mixed with different streptavidin (SAv)-fluorochrome combinations as detailed below at a 4:1 molar (pMHC-I monomer:SAv) ratio. Following multimerization, individual pMHC-I multimers were supplemented with 25 µM D-biotin (Sigma-Aldrich) to block free binding sites, 2 mM EDTA (Sigma-Aldrich), 2% FCS (Gibco), 5% Horizon™ Brilliant Stain Buffer Plus (BD Bioscience) and 100 nM dasatinib (Sigma-Aldrich) and were incubated for 20 min at 4°C.

pMHC-I multimer binding to TCR^+^ J76^CD8^ cells was analyzed by dsSCD and dtSCT multimerized through addition of SAv-R-phycoerythrin (SAv-PE, Miltenyi 130-106-790). Dual color-encoded pMHC-I libraries for the parallel interrogation of multiple CD8^+^ T cell epitopes in one staining experiment were prepared as described previously (3) with modifications. Defined pMHC-I monomers were multimerized with a unique SAv dual color combination and were finally pooled into a single pMHC-I library comprising SAv-PE (Miltenyi), SAv-allophycocyanin (SAv-APC, BioLegend 405207), SAv-Brilliant Violet (BV421, BD Bioscience 563259), SAv-BV711 (BD Bioscience 563262) and SAv-Brilliant Ultra Violet 395 (SAv-BUV395, BD Bioscience 564176) for the analysis of up to 10 antigen-specific T cell populations. For the multiplex analysis of up to 60 antigen-specific T cell populations at once, pMHC-I libraries were prepared using additionally SAv-BUV661 (BD 612979), SAv-BUV737 (BD 612775), SAv-PE-CF594 (BD 562284), SAv-PE-Cy5 (BioLegend 405205), SAv-PE-Cy7 (BioLegend 405206), SAv-BV785 (BioLegend 405249) and SAv-KIRAVIA Blue 520 (BioLegend 405172) omitting PE, PE-CF594, PE-Cy5 and PE-Cy7 dual color combinations.

For subsequent single-cell RNA sequencing (scRNA-Seq) analysis of DNA-barcoded cells, all identified pMHC-I multimer^+^ populations from the dual-color encoded analysis were pre-clustered into one of three groups for cell sorting depending on their frequencies (low (< 0.1%), intermediate (0.1%–1.0%) or high (>1%)) and associated with the fluorochromes APC, BV421 or BUV395, respectively, and then pooled in a defined ratio (also see **Supplementary Figure 2C** and **Supplementary Table 4**) to ensure that also populations of very low frequency were still detectable by the scRNA-Seq sample processing procedure with a limited targeted cell recovery of up to 10,000 cells.

A combined dual-colored and DNA-barcoded pMHC-I multimer library was generated compatible with 10x Genomics 5’ Single Cell RNA sequencing protocols with feature barcode technology, by adding 1.2 µl DNA-barcoded, SAv-PE-conjugated dextran polymer backbone (U-Load dCODE Dextramer^®^, Immudex^®^) to 3 µl of dtSCT, easYmer or peptide-loaded dsSCD diluted to 2 µM and was further supplemented with 1 µl U-Load dCODE Dilution Buffer (Immudex^®^). The same pMHC-I monomer was conjugated in parallel either with SAv-APC, SAv-BV421 or SAv-BUV395 based on the previously determined frequencies of dual-color encoded pMHC-I multimer^+^ CD8^+^ T cells from the melanoma patient. DNA-barcoded PE-pMHC-I multimer and corresponding pMHC-I multimer based on SAv-APC, SAv-BV421 or SAv-BUV395 were subsequently pooled into a single pMHC-I library before cell labeling and cell sorting.

### 2.11 pMHC-I multimer cell staining and cell sorting

Cultured TCR^+^ J76^CD8^ cells were washed once with pMHC staining buffer (DPBS supplemented with 2% FCS, 2 mM EDTA, 100 nM dasatinib) and were then stained with PE-labeled pMHC-I multimers diluted to approximately 50 nM in pMHC staining buffer at RT for 25 min. After pMHC-I multimer staining, TCR^+^ J76^CD8^ cells were washed once with pMHC staining buffer followed by labeling with murine TCR-Cβ-APC (clone H57-597, BioLegend) and CD8-PacificBlue (Clone SK1, BioLegend).

Cultured *ex vivo* CD8^+^ T cells were washed once with DPBS/100 nM dasatinib and were then labeled with the Zombie Aqua™ Fixable Viability Kit (BioLegend 423102) 1:300 for 10 min at RT in DPBS + 100 nM dasatinib. Next, one volume of pMHC staining buffer + Human TruStain FcX (Fc receptor blocking solution, BioLegend 422302) was added 1:50 (v/v) following an incubation for 5 min at RT. Cells were then stained with prepared pMHC-I multimer libraries at RT for 25 min. After one wash, cells were stained using a cocktail containing optimally titrated antibodies (all from BioLegend) against human CD14 (M5E2, Cat. No. 301842), CD16 (3G8, 302048), CD19 (H1B19, 302242), and CD335 (9E2, 331924) (all Brilliant Violet 510-conjugated, defined as dump channel); CD8 (SK1, BioLegend 344714) APC-Cy7, and CD3 (UCHT-1) Alexa Fluor 700 (BioLegend 300424).

For the labeling of CD8^+^ T cells in freshly isolated PBMC with a DNA-barcoded pMHC-I multimer library, 0.1 µg/ml herring sperm DNA (Invitrogen™) was additionally added to the pMHC-I staining buffer and the above-mentioned antibody panel was appended by an antibody mix containing 30 DNA-barcoded TotalSeq™-C antibodies (BioLegend) as listed in **Supplementary Table 4**.

Finally, the stained TCR^+^ J76^CD8^ cells or PBMC-derived CD8^+^ T cells were stored in DPBS supplemented with 2.5% (v/v) paraformaldehyde and 1% FCS before flow cytometry measurement on a LSRFortessa flow cytometer and analyzed according to the gating strategy shown in **Supplementary Figure 2** using FlowJo (BD Biosciences) v.10.9.0. In the dual color-encoded pMHC-I multimer-binding data shown, pMHC-I multimer binding CD8^+^ T cells were identified by a Boolean gating strategy as live CD8^+^ T cells stained positive in two pMHC multimer channels and negative in all other pMHC multimer color channels, as previously described (50, 51).

CD8^+^ T cells labeled with the DNA-barcoded pMHC-I multimer library were kept in pMHC staining buffer and pMHC-I multimer positive cells were sorted with a FACSAria™ Fusion cell sorter (BD Biosciences) according to the gating strategy shown in **Supplementary Figure 2** into tubes containing 200 μl pMHC staining buffer.

### 2.12 Single-cell RNA sequencing of pMHC-I multimer^+^ CD8^+^ T cells

Sorted DNA-barcoded pMHC-I multimer^+^ CD8^+^ T cells of the melanoma patient were analyzed by single cell RNA sequencing (scRNA-Seq) utilizing the Chromium NEXT GEM Single Cell 5′ TCR profiling and Feature Barcode Technology v2 (dual index) reagent kit (10x Genomics), which enables the combined interrogation of cell surface protein expression including pMHC-I multimer binding (CSP), TCR (VDJ) and gene expression (GEX). Cells were processed according to instructions by 10x Genomics (Protocol CG000330 Rev D). Fourteen cycles of initial cDNA amplification were used for all sets and single-cell sequencing libraries for whole-transcriptome analysis (GEX), TCR profiling (VDJ), and combined cell-surface protein and dCODE Dextramers detection (CSP) were generated. Libraries were quality controlled by automated gel electrophoresis (Agilent TapeStation) and quantified using a Qubit Fluorometer (Thermo Scientific), and finally pooled in a ratio of 4:1:1 (GEX:VDJ:CSP) and sequenced on a NextSeq 550 system (Illumina) using 150 cycles on the basis of sequencing by synthesis (SBS) chemistry with cycle configuration (read 1: 26 bp; index read 1: 10 bp; read 2: 90 bp), with a sequencing depths of at least 20000, 5000, 5000 reads pairs per cell for the GEX, VDJ, CSP libraries, respectively.

Raw scRNA-seq FASTQ files were aligned to the human GRCh38 genome with Cell Ranger version 7.1.0 with default settings for the ‘cellranger multi’ pipeline (10x Genomics) for combined V(D)J, gene expression and antibody capture (cell surface protein) analysis and GEX:VDJ:CSP libraries were paired for downstream assessment of the data set. The Loupe Cell Browser version 7.0.0 (10x Genomics) software was used for data analysis including cell clustering and data visualization.

### 2.13 Mutation identification and neoepitope prediction

A tumor biopsy sample of lymph node metastasis of a melanoma patient and matching PBMC sample was sequenced by the DKFZ GPCF as part of the MASTER program (52) to identify expressed somatic nucleotide variants (SNV), genetic insertions and deletions (InDels) and gene fusion events. Whole exome sequencing (WES) of DNA libraries was done using a on a NovaSeq 6000 system (Illumina) (2x 100 bp) and demultiplexing of the sequencing reads was performed with Illumina bcl2fastq (2.20). Adapters were trimmed with Skewer (version 0.2.2) (53). Alignment of sequencing reads was done by the DKFZ alignment workflow from the ICGC Pan-Cancer Analysis of Whole Genome projects (DKFZ AlignmentAndQCWorkflows v1.2.73, (https://github.com/DKFZ-ODCF/AlignmentAndQCWorkflows). The human reference genome version GRCh37/hg19 was used. RNA libraries from the tumor biopsy were prepared using the Kapa RNA HyperPrep Kit with RiboErase (Roche) and subjected to a NovaSeq 6000 system for RNA sequencing (RNA-Seq). RNA-Seq reads were aligned and gene expression quantified using the DKFZ RNA-Seq (v1.2.22-6, https://github.com/DKFZ-ODCF/RNAseqWorkflow). For total library abundance calculations, during TPM and FPKM expression values estimation, genes on chromosomes X, Y, MT, and rRNA and tRNA were omitted to avoid library size estimation biases as previously described (54, 55). SNV and InDel mutation calling was done using DKFZ in-house piplines (SNVCallingWorkflow v1.2.166-1, https://github.com/DKFZ-ODCF/SNVCallingWorkflow); IndelCallingWorkflow v1.2.177, https://github.com/DKFZ-ODCF/IndelCallingWorkflow) as previously described (56). Raw calls for InDels were obtained from Platypus (57). The proteins coding effect of SNVs and InDels were annotated using ANNOVAR according to GENCODE gene annotation (version 19) (58) and overlapped with variants from dbSNP10 (build 141) and the 1000 Genomes Project database. Mutations of interest were defined as somatic SNV and InDels that were predicted to cause protein coding changes (non-synonymous SNVs, gain or loss of stop codons, splice site mutations, frameshift and non-frameshift indels) (55). Gene fusion events were detected by applying the Arriba algorithm (59) on the RNA-Seq data set. Neoepitopes were predicted from raw sequencing data by a comprehensive and fully automated DKFZ in-house pipeline (60), which is implemented in an Anaconda environment to ensure easy usage and reproducibility. The pipeline integrates previously identified SNVs, InDels, gene fusion events as well as the gene expression level and generates mutations sequences that are flanked by 10 wildtype amino acids upstream and downstream for an SNV mutation and 10 wildtype amino acids upstream of frameshift mutations. Mutated protein sequences were finally queried by the netMHCpan 4.1 algorithm (61) to predict potential binding and presentation by patient’s HLA-I alleles. 166 unique peptide/HLA neoepitope candidates with a predicted %Rank_EL > 2.5 spanning 49 SNV and 2 gene fusion events were selected for dsSCD and easYmer binding analysis and subsequent immunogenicity screening.

### 2.14 Statistical analysis

Unless otherwise stated, all results are expressed as mean ± SD. Analysis and graphical representations were conducted using GraphPad Prism 8 software (GraphPad Software Inc.). Experiments containing more than 2 experimental groups were analyzed using a one- or two-way analysis of variance (ANOVA) with Tukey’s multiple comparison test. The number of donors and experiments, as well as the statistical analysis is stated in the respective figure legends with p values <0.05 considered statistically significant (ns, *p*>0.05; *, *p*<0.05; **, *p*<0.01; ***, *p*<0.001; ****, *p*<0.0001).

## 3 Results

### 3.1 Peptide-receptive empty MHC-I dsSCD produced in mammalian cells

**Figure 1:**
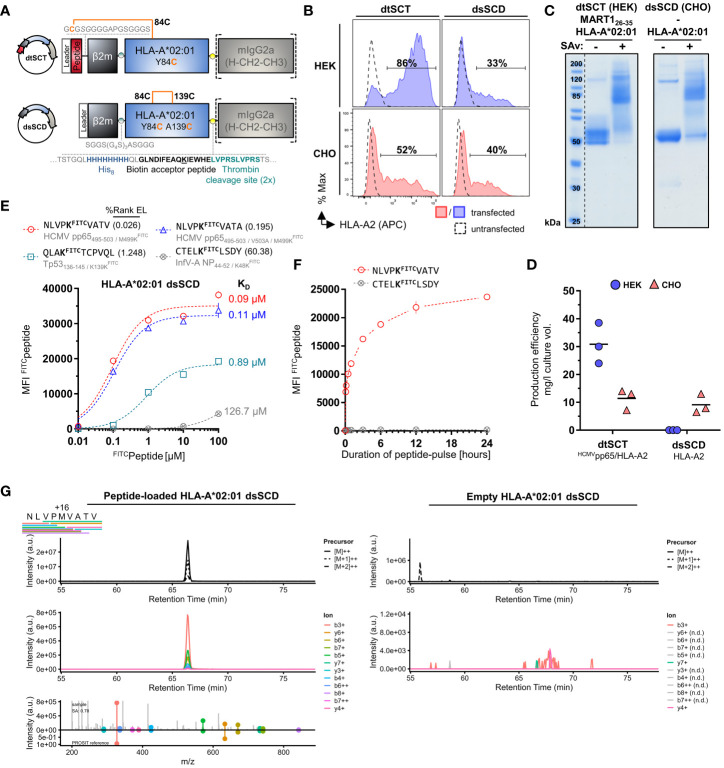
Successful CHO cell-based production of *in vivo* biotinylated, empty, peptide-receptive, disulfide-stabilized β_2_m-HLA-A*02:01 single-chain dimers (dsSCD). **(A)** Schematic representation of soluble disulfide-trapped peptide-β_2_m-HLA-A*02:01 single-chain trimer (dtSCT) (upper panel) and dsSCD (lower panel). dtSCT consist of a single polypeptide chain comprising a peptide ligand, human β_2_-microglobulin (β_2_m) and MHC-I ectodomain that are covalently linked via flexible glycine-serine-rich linkers (GSL). The SCT intramolecular disulfide trap is generated between a cysteine [C] in the first GSL and a C residue replacing a conserved MHC-I tyrosine [Y] residue at position 84. In dsSCD, β_2_m is fused *via* a GSL to the MHC-I ectodomain that harbors an additional alanine [A] A139C, Y84C disulfide bridge that stabilizes the β_2_m-MHC-I complex also in the absence of a peptide ligand. dtSCT and dsSCD are C-terminally fused to an octa-histidine tag (His_8_), a biotin acceptor peptide that is followed by two consecutive (2x) thrombin cleavage sites fused to a murine IgG2a Fc part. All constructs are designed for mammalian cell expression and were always transiently co-transfected with ER-retained Igκ_SP_-BirA_KDEL_ biotin ligase to allow for site-specific biotinylation during dsSCD/dtSCT expression. **(B)** Representative intracellular HLA-A*02:01 dtSCT and dsSCD expression analysis by flow cytometry 48 hours post plasmid (filled lines) and mock (dotted line) transfection of HEK293-F (HEK) and CHO-S (CHO) cells using anti-HLA-A2 antibody BB7.2. **(C)** SDS-PAGE analysis of affinity chromatography-purified monomeric dtSCT and dsSCD from HEK and CHO cell supernatants, respectively, that have been treated with thrombin to cleave the Fc portion. DtSCT and dsSCD biotinylation is confirmed by equimolar addition of streptavidin prior to SDS-PAGE analysis leading to a gel shift. **(D)** Production efficiencies of dtSCT and dsSCD expressed in HEK or CHO. Values were calculated using the yield in mg of the final protein yield divided by the culture supernatant volume in liters used for affinity chromatography. Dots represent three independent cell transfections for the conditions shown. **(E, F)** Flow cytometry-based FITC-conjugated peptide (^FITC^peptide) binding assay for bead-immobilized dsSCD. FITC median fluorescence intensities (MFI) of ^FITC^peptide-loaded dsSCD immobilized on streptavidin-conjugated beads in triplicates are shown. HLA-A*02:01 dsSCD beads were pulsed with the indicated concentrations of ^FITC^peptides **(E)**, or by a ^FITC^peptide pulse at 1 µM for the indicated time periods starting with 5, 10, 30 and 60 minutes and ending with 24 hours (h) **(F)**. HLA-A*02:01 %Rank EL prediction values are shown for the indicated peptides with K substitution and their binding levels as putative strong binders (SB, %Rank EL <0.5), weak binders (WB, %Rank EL <2) and non-binders (nonB, %Rank >2) according to the NetMHCpan 4.1 algorithm. The FITC conjugation of the peptide is neglected in this assessment. **(E)** Non-linear regression (one-site specific binding) of the FITC MFI values from means of triplicates (± SD) against the peptide concentration and calculated K_D_ values in µM are shown. **(F)** Non-linear regression of the FITC MFI from means of triplicates (± SD) against the incubation time is shown. **(G)** Data-independent acquisition mass spectrometry (DIA-MS) of NLVPMVATV loaded (left panels) and empty (right panels) HLA-A*02:01 dsSCD. Left panels show the manual detection of the NLVPMVATV peptide eluted from the externally peptide-loaded HLA-A*02:01 dsSCD. Bars below the NLVPMVATV sequence indicate detected fragment ions in peptide-loaded sample. Top panels show extracted ion chromatograms (XICs) of the precursor and its isotopes, middle panels shows XICs of fragment ions. The top half of the bottom panel shows the MS2 spectra extracted at the highest point of the MS2 XIC, while the bottom half shows the mirrored spectrum as *in silico* predicted by PROSIT. The spectral angle (SA) was calculated and is indicated. Right panels show the lack of matching precursor and fragment ions for the empty dsSCD molecule within a 25 min window centered around the retention time of NLVPMVATV peptide. n.d, not detected.

For the generation and purification of monomeric pMHC-I dtSCT that can be multimerized by streptavidin (SAv) we modified our previously reported Fc-tagged dtSCT format (25) by introducing a His_8_ tag, a biotin acceptor peptide, and a tandem thrombin cleavage site between the MHC-I ectodomain and mIgG2a Fc portion (**Figure 1A**). Fc-tagged dtSCT were produced in HEK293 cells co-transfected with secretory BirA biotin ligase retained by a C-terminal KDEL ER retention signal (62). Treatment of cell supernatants with thrombin liberated biotinylated dtSCT monomers (ca. 57 kDa) as shown exemplarily in **Figure 1C** for a MART1_26-35_/HLA-A*02:01 dtSCT, that produced a gel shift after incubation with streptavidin indicating SDS-stable complex formation with 2-4 streptavidin monomers of ~15 kDa.

To generate a dsSCD, the peptide sequence and first flexible linker was omitted and in addition to the mutation Y84C present in dtSCT, the mutation A139C was introduced in the MHC-I α2 domain to enable the formation of an artificial intramolecular disulfide bond stabilizing the C-terminal end of the peptide binding groove (9, 10). In contrast to dtSCT that were efficiently produced, HLA-A*02:01 dsSCD were not secreted in detectable amounts by HEK293-F cells albeit being expressed intracellularly (**Figures 1B, D**). Therefore, we replaced HEK293-F cells by CHO-S producer cells leading to satisfying yields of secreted dsSCD in the range of 10 mg/l culture volume (**Figure 1D**). DsSCD produced in CHO-S cells were, however, only partially biotinylated by co-expressed BirA ligase as indicated by partial gel shifts (dsSCD: ca. 55 kDa after thrombin cleavage, **Figure 1C**). With similar production yields, a large series of dsSCD allotypes lacking the thrombin cleavage site and Fc portion were produced in CHO-S cells (**Supplementary Figure 1A**, **Supplementary Table 1**). These shortened variants avoid the potential problem of incomplete thrombin cleavage.

We next analyzed the capacity of bead-immobilized HLA-A*02:01 dsSCD to bind three known HLA-A*02:01 ligands and one predicted non-binder by flow cytometry using fluorescently modified variants thereof containing a Lys^FITC^ residue (**Figure 1E**). While two peptides (NLVPK^FITC^VATV and NLVPK^FITC^VATA), *in silico* predicted in their non-FITC-conjugated forms to be high affinity binders, reached saturation plateau signals at 1 µM, at least 10 µM were required for a predicted low affinity binder (QLAK^FITC^TCPVQL), while the predicted non-binder (CTELK^FITC^LSDY) only showed minimal binding at 100 µM after incubating for 18 h at ambient temperature. To allow low affinity peptides to occupy dsSCD at least partially, 10 µM or higher was chosen as the standard concentration for overnight loading of unlabeled test peptides. **Figure 1F** shows the association kinetics of the Lys^FITC^-substituted high affinity binder, HCMV pp65_495-503_, at 1 µM concentration to bead-immobilized HLA-A*02:01 dsSCD at ambient temperature in comparison to a non-binder, a known HLA-A*01:01 ligand. Between 12 and 24 h of incubation, binding of a the Lys^FITC^-substituted NLV peptide reached saturating levels.

Mass-spectrometry (MS) was used to examine whether HLA-A*02:01 dsSCD produced in CHO-S cells were loaded with endogenous peptides or not, and whether peptide ligands loaded as positive controls could be re-identified. No peptides of hamster origin could be identified using data-independent acquisition MS, indicating that dsSCD molecules were *bona fide* free of peptide after purification. After loading with a peptide pool of known high-affinity A*02:01 ligands, acid-eluted peptides corresponding to expected binders could be re-identified by MS, e.g. HCMV pp65_495-503_ (NLVPMVATV) (**Figure 1G**, left panels), while the data generated from untreated dsSCD was devoid of a corresponding peptide signal at the respective retention time window (**Figure 1G**, right panels and data not shown).

**Figure 2:**
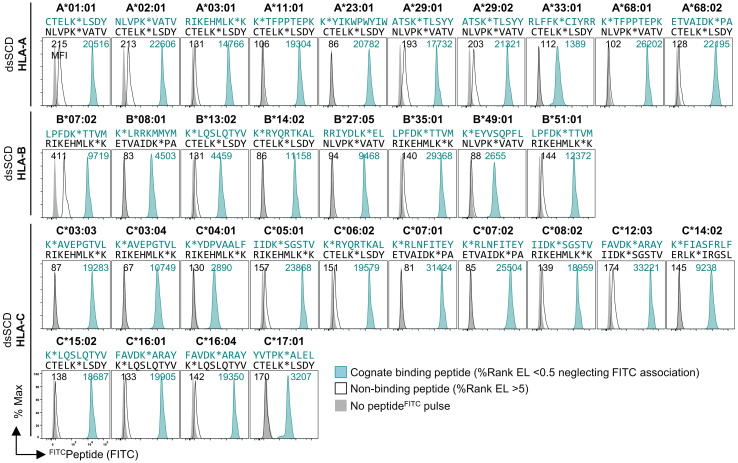
Validation of 32 produced HLA-A, -B and -C dsSCD by ^FITC^peptide binding assay. Individual production yields of dsSCD allotypes and used ^FITC^peptides are listed in **Supplementary Table 1**. For dsSCD testing, a set of peptides were rationally designed to have at a selected non-anchor residue a K^FITC^ substitution (K*) based on known ligands, the NetMHCpan 4.1 binding motif viewer and %Rank EL values for the indicated HLA allotype. FACS histograms are shown for bead-immobilized dsSCD pulsed for 18 h with 1 µM of a binding ^FITC^peptide (peptide sequence and plot shown in teal) and non/poor binding ^FITC^peptide (black line) and bead background MFI (gray filled) for the indicated HLA allotypes.

To obtain more information about the binding properties of Lys^FITC^ substituted peptides, we compared the HCMV pp65_495-503_ high-affinity peptide, NLVPMVATV (N9V), substituted in all 9 positions for Lys^FITC^, for binding to bead-immobilized HLA-A*02:01 dsSCD (**Supplementary Figure 1B**). Equilibrium dissociation constants (K_D_ values) that were calculated for observed binding curves show that the bulky Lys^FITC^ side chain was not tolerated at the major anchor positions P2 and P9 and to a minor extent also at positions P4 and P6. For comparison we show the NetMHCpan motif for HLA-A*02:01 and *in silico* predicted relative binding probabilities for Lys-substituted N9V homologs with predicted strong binders ≤ 0.5% Rank_EL and weak binders ≤ 2.0% Rank_EL. We conclude that Lys^FITC^-modified indicator peptides mirror the peptide binding properties of known HLA-A*02:01 ligands sufficiently well and can be used as competitors in peptide binding assays with dsSCD if non-anchor positions are substituted. In a similar fashion we rationally designed Lys^FITC^-modified indicator peptides, based on known strong binders and considering only non-anchor positions for substitution, for a total of 32 dsSCD HLA-A,B,C allotypes presented in this work. We demonstrate successful binding to bead-immobilized respective dsSCD by flow cytometry (**Figure 2**, **Supplementary Table 1**).

### 3.2 Antigen-specific T cell detection with dsSCD-based multimers

**Figure 3:**
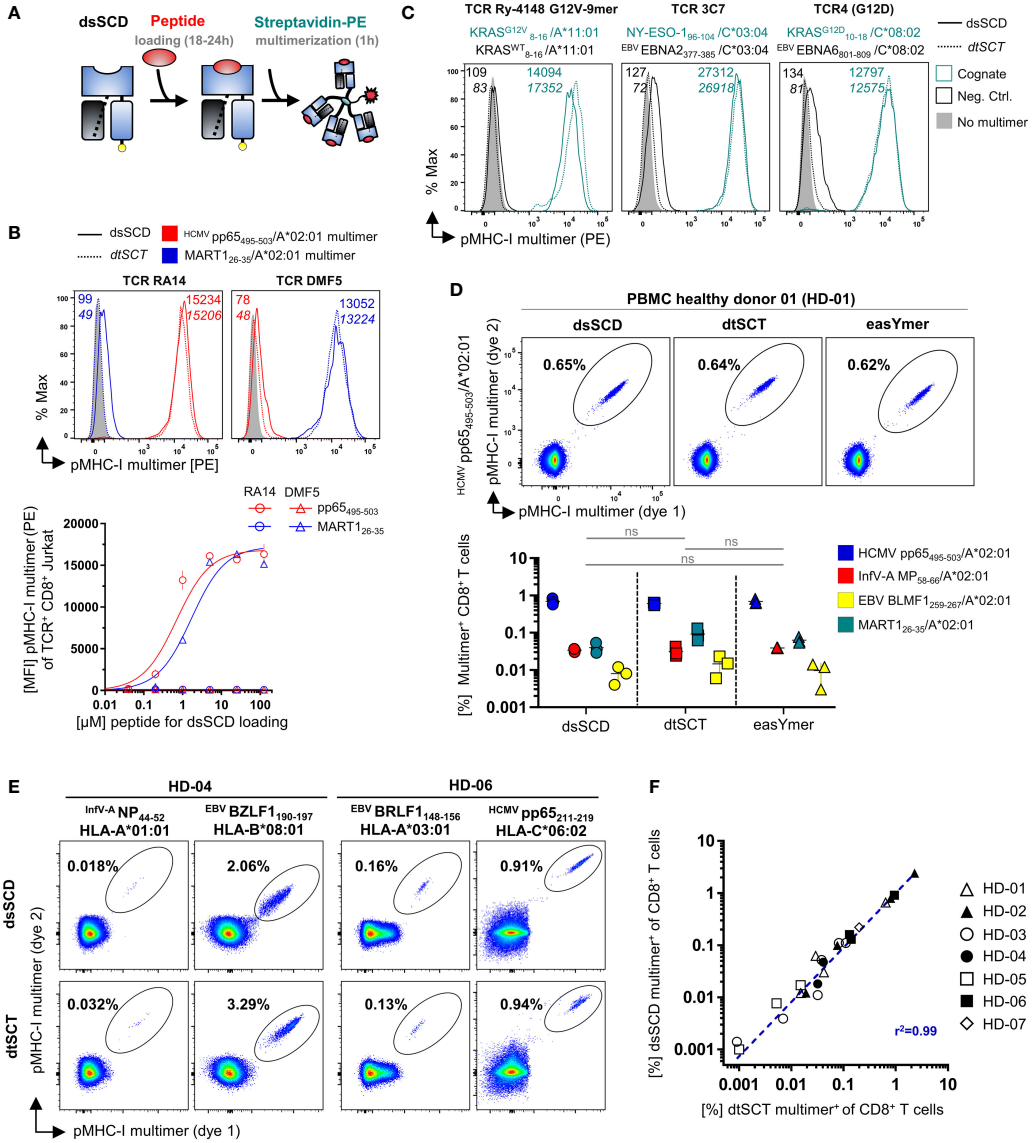
Comparable antigen-specific labeling of TCR-transgenic CD8^+^ Jurkat and healthy donor-derived viral epitope-specific CD8^+^ T cell populations with peptide-loaded dsSCD and dtSCT multimers. **(A)** Schematic representation of dsSCD for the usage as antigen-specific labeling reagent for defined CD8^+^ T cell populations. Following an overnight peptide pulse, the biotinylated dsSCD are multimerized by the addition of a fluorochrome-labeled streptavidin. **(B, C)** Cognate dtSCD and dsSCD multimer labeling of CD8^+^ Jurkat 76 (J76^CD8+^) stably expressing various published TCR as recombinant chimeric TCR containing murine Cβ and Cα domains. **(B)** J76^CD8+^ cells stably expressing RA14 TCR recognizing HLA-A*02:01/^HCMV^pp65_495-503_ or DMF5 TCR recognizing HLA-A*02:01/MART-1_26-35_ were labeled with HLA-A*02:01 dsSCD previously loaded at varying concentrations with peptide ^HCMV^pp65_495-503_ (red symbols) or MART-1_26-35_ (blue symbols), respectively, for 18 h followed by multimerization. The upper panel shows representative histograms of RA14 and DMF5 J76^CD8+^ labeled with dsSCD multimers loaded with 25 µM peptide (filled line, MFI values in plain font) or dtSCT multimers (dotted line, MFI values in *italics*). PE fluorescence-minus-one (FMO) controls of J76^CD8+^ are shown in filled gray. The lower panel shows RA14 (circles) and DMF5 (triangles) J76^CD8+^ staining efficiencies at varying peptide loading concentrations used for dsSCD loading in triplicates and fitting using non-linear regression. For control, RA14 and DMF5 J76^CD8+^ cells were stained with dsSCD loaded with the non-cognate peptide. **(C)** Antigen-specific staining of published neoepitope or tumor-associated antigen-specific HLA-A*11:01, HLA-C*03:04 and HLA-C*08:02-restricted TCR with multimerized dtSCT and dsSCD harboring the cognate peptide (teal) or a dsSCD/dtSCT-binding control peptide (black). **(D, E)** Detection of virus-specific human CD8^+^ T cell populations in healthy donors with dsSCD and dtSCT multimers using combinatorial dual-fluorochrome encoding. **(D)** Labeling of HLA-A*02:01^+^ healthy donor 01 (HD-01) CD8^+^ T cells from PBMC with HLA-A*02:01 multimers generated on the basis of dsSCD, dtSCT or the commercial easYmer. The upper panel shows a representative dot plot of a ^HCMV^pp65_495-503_/HLA-A*02:01-specific CD8^+^ T cell population. The lower panel shows the identification of four different HLA-A*02:01-restricted multimer^+^ populations found in three independent experiments across all three tested pMHC-I multimer platforms. The data set was statistically analyzed by two-way ANOVA followed by Tukey’s multiple comparison test and no significant (ns) differences between the platforms were found. **(E)** Labeling of multiple HLA-typed healthy donors with pairs of dtSCT and dsSCD multimers representing 16 known viral and tumor-associated epitopes. Multimer^+^ populations using HLA-A*01:01, A*03:01, B*08:01 or C*06:02 dtSCT and dsSCD in HD-04 and HD-06 are exemplarily shown. **(F)** Correlation analysis of antigen-specific T cell frequencies detected by HLA-A*01:01, A*02:01, A*03:01 or C*06:02 dsSCD and dtSCT multimers in PBMC-derived CD8^+^ T cells of HD-01–07. The individual T cell frequencies and specificities are listed in **Supplementary Table 2**.

We examined the capacity of biotinylated peptide-loaded dsSCD to assemble with fluorochrome-labeled streptavidin (SAv) as MHC-I multimers and stain antigen-specific T cells. To form pMHC-I multimer reagents, monomeric dsSCD were loaded with appropriate peptides for 18-24 h and subsequently multimerized with fluorochrome-labeled streptavidin (**Figure 3A**). As proof of concept, HLA-A*02:01 dsSCD were loaded with peptides HCMV pp65_495-503_ or MART1_26-35_, complexed with PE-conjugated SAv and used for the staining of CD8^+^ Jurkat 76 cells transfected with TCRs RA14 or DMF5, respectively. For comparison, multimers of biotinylated dtSCT containing the same peptides in tethered form were used in parallel. Both peptide-loaded dsSCD and dtSCT homogenously stained Jurkat 76/CD8 transfectants with equal efficiencies (**Figure 3B**). A titration of peptides used for the loading of HLA-A*02:01 dsSCD showed that 25 µM is a suitable concentration to achieve maximal labeling of TCR-transfected Jurkat cells (**Figure 3B**). In **Figure 3C** we show the comparative stainings of TCR-transfected Jurkat cells with peptide-loaded dsSCD and dtSCT multimers representing complexes of HLA-A*11:01 or HLA-C*08:02 with mutant KRAS peptides, as well as HLA-C*03:04 presenting an NY-ESO-1 peptide. Again, peptide-loaded dsSCD and dtSCT performed equally well. The labeling was highly efficient and peptide-specific, as TCR Ry-4148 did not bind dsSCD and dtSCT loaded with a KRAS wild-type control peptide. Since the KRAS G12D mutation in peptide GA**D**GVGKSA represents a neo-anchor for HLA-C*08:02 and the respective wild-type peptide did not bind to dsSCD nor allowed the production of a respective dtSCT, an unrelated EBNA6 peptide was used as negative control.

To compare the staining performances of the peptide-loadable dsSCD (MediMer) and peptide-tethered dtSCT platforms with the commercially available easYmer platform (immunAware) based on *in vitro* folding of wild-type MHC heavy chain into a stable peptide–β_2_m–MHC-I complex (7), the individual platforms were multimerized in parallel and applied in dual-color encoded pMHC-I multimer stainings for the detection of HCMV pp65, EBV BMLF1, Influenza-A MP and MART1 antigen-specific CD8^+^ T cells population of a HLA-A*02:01 positive healthy donor (**Figure 3D**). In the top panel, analysis of pMHC-I multimer stainings of pp65-specific T cells from donor HD-01 are exemplarily shown based on a Boolean gating strategy as explained in **Supplementary Figure 2**. Detected population frequencies of HD-01 determined in three independent experiments using the three pMHC-I platforms (bottom panel) revealed equal efficacies to detect the 4 analyzed T cell populations ranging between 0.01% and 1% in size. Proof-of-concept stainings with HLA-A*02:01 dsSCD multimers were then extended to various additional viral T cell epitopes restricted by HLA-A*01:01, HLA-A*03:01 and HLA-B*08:01 and HLA-C*06:02 detected in the peripheral blood of HLA-typed healthy donors as exemplarily shown for HD-04 and HD-06 (**Figure 3E**). Respective dsSCD allotypes were loaded with synthetic peptides and compared with dtSCT carrying the same peptides in tethered form. Again, similar frequencies of multimer-stained T cells were detected with both reagents across various HLA-A, -B, -C allotypes. The results from comparative dsSCD and dtSCT multimer stainings addressing 16 viral and tumor-associated epitopes recognized by CD8^+^ T cells in peripheral blood from 7 different healthy donors are summarized in the correlation plot of **Figure 3F**, showing a highly consistent detection of T cell populations by both multimer tools over a large range of frequencies (see **Supplementary Table 2** for details of peptides and restricted HLA-I allotypes).

### 3.3 Empty and peptide-loaded dsSCD are functionally stable due to tethered β_2_m

**Figure 4:**
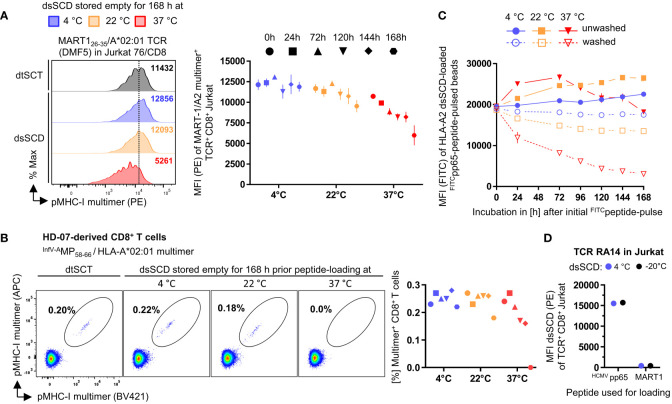
Stability assessment of empty and peptide-loaded dsSCD. **(A, B)** Empty dsSCD were stored for up to one week at 4°C, 22°C or 37°C followed by a peptide pulse with MART-1_26-35_ or ^InfV-A^MP_58-66_ peptide and multimerization with streptavidin-PE. The shown durations and storage temperatures indicate conditions used for empty dsSCDs storage before dsSCD were finally loaded with 10 µM peptide and then kept at the indicated temperature peptide-loaded until 168 h were completed. To this add ~3 h incubation time until multimerization was accomplished and samples were analyzed by flow cytometry. Temperature-challenged dsSCD multimers, and dtSCT-based multimers serving as positive controls, were used to stain DMF5 TCR-transfected J76^CD8+^ **(A)** and healthy donor 07 (HD-07)-derived CD8^+^ T cells **(B)**. **(A)** Representative histogram of DMF5 TCR J76^CD8+^ labeled with multimerized dsSCD that have been stored empty for 168 h at various temperatures (left panel) and MFI values of the entire experiment (right panel). **(B)** Staining of HD-07 CD8^+^ T cells with multimerized ^InfV-A^MP_58-66/_HLA-A*02:01 dsSCD stored empty for 168 h at various temperatures (left panel) and MFI values of the entire experiment (right panel). Data represent single values. **(C)** Dissociation analysis of ^FITC^peptide-loaded dsSCD. Beads with immobilized HLA-A*02:01 dsSCD were pulsed overnight with 1 µM NLVPK^FITC^VATV at 4°C in PBS and were washed with PBS (dotted line) or left unwashed (solid line) at the indicated temperatures and incubation times followed by flow cytometric analysis of the remaining FITC MFI. The 0 h values represent the MFI baseline measured after the initial 24 h peptide pulse. **(D)** Analysis of the freezing compatibility of dsSCD. Empty HLA-A*02:01 dsSCD were stored in the presence of glycerol and BSA at -20°C (black dots) or left in PBS at 4°C (blue dots) for 3 weeks before usage as cognate or control peptide-loaded dsSCD multimers for the staining of HCMV pp65-reactive RA14 TCR-transfected J76^CD8+^ cells. In **(A, C, D)**, the shown data represent the MFI values ± SD of the means of 3 technical replicates.

To validate the robustness of peptide-receptive dsSCD for the laboratory praxis, we next analyzed the stability of peptide-free and peptide-loaded dsSCD. Purified HLA-A*02:01 dsSCD were stored empty under sterile conditions for the indicated time periods up to one week at 4, 22 or 37°C and were left loaded with 10 µM MART1_26-35_ peptide for the remaining time at the indicated temperatures before being finally used for the staining of TCR DMF5-transfected Jurkat 76/CD8 cells (**Figure 4A**). After keeping empty or peptide-loaded dsSCD at 4°C for one week the staining capacity was unaltered and very similar to a MART1/A*02:01 dtSCT-based staining. After storing empty dsSCD longer than 120 h at ambient temperature, the staining capacity was slightly diminished, while after storage at 37°C for up to one week the staining was reduced to about 50% suggesting that dsSCD molecules had slowly deteriorated leading to reduced peptide receptivity. In a parallel experiment, influenzavirus A matrix protein_58-66_ peptide-loaded HLA-A*02:01 dsSCD stored at various conditions were used to detect a small population of MP-reactive T cells in the peripheral blood of a healthy donor (**Figure 4B**). In accordance with previous results, storage of empty dsSCD for one week at 37°C led to a complete loss of multimer staining while storage of empty or peptide-loaded dsSCD at 4°C did not impair peptide receptivity or subsequent pMHC-I multimer stainings, respectively. While a systematic stability analysis of empty dsSCD was only conducted with the HLA-A*02:01 allotype, here we report the observation that empty dsSCD of various HLA-A,B,C allotypes exhibited unimpaired peptide receptivity after storage at 4°C for 3-12 months (data not shown).

We also assessed the stability of peptide association with dsSCD at various temperatures. To this end, bead-immobilized dsSCD were initially loaded overnight with 1 µM HCMV pp65 N9V[M>K^FITC^] peptide at 4°C before incubating the peptide-loaded dsSCD for different time periods up to one week at 4, 22 or 37°C and measuring fluorescence by flow cytometry (**Figure 4C**). After the initial overnight peptide pulse, beads were either washed to remove unbound peptide or kept in the presence of peptide to keep peptide binding at equilibrium. In the presence of excess peptide, dsSCD continued to slowly take up additional peptide which was favored by incubation at ambient temperature, whereas the peptide receptivity of dsSCD incubated at 37°C began to slowly decay after 3 days. Washed dsSCD-N9V^FITC^ complexes bound to beads were fully stable at 4°C, slightly decayed at 22°C, yet rapidly dissociated when incubated at 37°C.

For routine usage it would be useful if dsSCD can be stored frozen without loss of function. To analyze this question, HLA-A*02:01 dsSCD were frozen in glycerol-containing buffer at -20°C for 3 weeks before thawing and charging with unlabeled HCMV pp65_495-503_ peptide or MART1_26-35_ control peptide (**Figure 4D**). Respective pMHC-I multimers were used to stain TCR RA14-transfected J76^CD8+^ cells in comparison to multimers formed with dsSCD that had been stored at 4°C. Defrozen empty dsSCD molecules fully retained their capacity to load peptide, form multimers and stain peptide-specific T cells, yet also did not gain unspecific binding due to aggregation when loaded with the irrelevant MART1 peptide.

**Figure 5:**
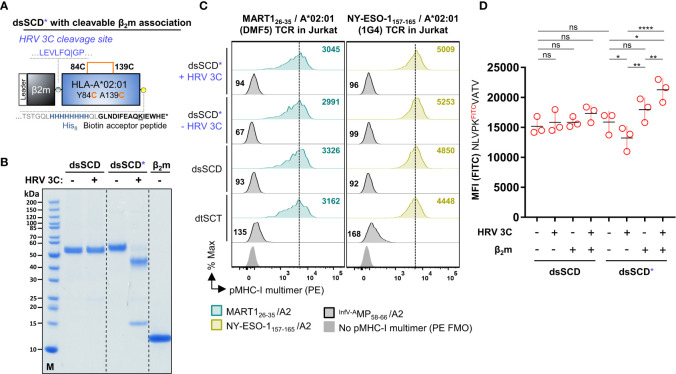
Analysis of HLA-A*02:01 dsSCD with cleavable β2m linker. **(A)** Schematic representation of a disulfide-stabilized β_2_m-HLA-A*02:01 single-chain dimer with an additional HRV 3C cleavage site at the C-terminal end of the glycine-serine linker between β2m and MHC-I ectodomain (dsSCD*). **(B)** SDS-PAGE analysis of affinity chromatography-purified monomeric HLA-A*02:01 with (dsSCD*) and without (dsSCD) HRV 3C cleavage site incubated overnight in the presence (+) or absence (–) of HRV 3C protease. After dsSCD* cleavage with HRV 3C, free linker-extended β2m is visible. For comparison human β_2_-microglobulin isolated from urine is shown. **(C)** Antigen-specific labeling of DMF5 and 1G4-TCR transgenic J76^CD8+^ cells with multimerized peptide-loaded dsSCD* with non-covalent β_2_m association (+ HRV 3C) and covalent β_2_m association (- HRV 3C). Here, dsSCD* were incubated with HRV 3C overnight followed by a consecutive peptide pulse (25 µM) and multimerization. A corresponding pMHC-I multimer staining using peptide-loaded dsSCD without HRV 3C cleavage site and dtSCT carrying the same peptides is additionally shown. **(D)** Analysis of the ^FITC^peptide-loading capacity of bead-immobilized dsSCD* and dsSCD treated with HRV 3C. HLA-A*02:01 dsSCD and dsSCD* were incubated overnight in the presence (+) or absence (–) of HRV 3C protease and additional supplementation with a 4.6-fold molar excess of free β2m (+) or no β2m (–) during this incubation step. Treated dsSCD and dsSCD* were immobilized on streptavidin beads and loaded with 1 µM NLVPK^FITC^VATV peptide overnight followed by flow cytometric analysis. Data represent mean values from 3 independent experiments in triplicates with statistical analysis by one-way ANOVA test followed by Tukey’s multiple comparison test. Error bars show the standard deviation. ns, not significant; *p< 0.05; **p< 0.01; ****p< 0.0001.

In contrast to soluble disulfide-stabilized MHC-I heavy chains that were refolded *in vitro* in the presence of an excess of free β_2_m (10), here we studied single-chain dimers in which the C-terminus of the β_2_m open reading frame is tethered to the N-terminus of a disulfide-stabilized MHC-I α1 domain through a 24 amino acid long flexible linker. Tethering is conceived to facilitate the re-assembly of dissociated β_2_m since interaction partners remain in close vicinity due to the linker. To study the influence of tethered β_2_m on the peptide receptivity of dsSCD, we introduced the human rhinovirus (HRV) 3C protease cleavage sequence (LEVLFN|GP) in an extended linker sequence between β_2_m and disulfide-stabilized HLA-A*02:01 heavy chain (**Figure 5A**). As intended, by addition of 3C protease to the β_2_m-cleavable dsSCD*, a dissociation of β_2_m from the MHC heavy chain could be visualized by SDS-PAGE analysis, whereas 3C protease treatment did not affect the apparent molecular weight of the non-cleavable dsSCD (**Figure 5B**). First we studied the staining capacity of pMHC-I multimers loaded with MART1 and NY-ESO-1 peptides for TCR-transfected Jurkat 76/CD8 cells. Peptide-loaded, HRV 3C-cleaved dsSCD* stained antigen-specific TCR as efficiently as non-cleaved dsSCD*, dsSCD or dtSCT-based multimers (**Figure 5C**), suggesting that a potential loss of peptide receptivity due to β_2_m cleavage remained below the threshold of detection. Alternatively, dsSCD with and without HRV 3C cleavage sequence were incubated for 18 h in the absence or presence of 3C protease and with or without a 4.6-fold molar excess of free human β_2_m. Treated dsSCD were then immobilized and loaded overnight with 1 µM N9V^FITC^ peptide (**Figure 5D**). Here, cleavage by 3C in the absence of additional β_2_m resulted in a significant reduction of the FITC signal suggesting a partial loss of the peptide receptivity after irreversible dissociation of cleaved β_2_m. An excess of free β_2_m rescued loading with N9V^FITC^ peptide to the full extent and even slightly enhanced peptide uptake, suggesting a slightly greater functionality of free β_2_m replacing tethered β_2_m, while addition of free β_2_m had no significant effect on non-cleavable dsSCD nor on uncleaved, cleavable dsSCD.

Taken together, biotinylated dsSCD that are purified from supernatants of CHO-S producer cells can be stably charged with synthetic peptides and used as MHC-I multimer tools similar to the established easYmer platform and biotinylated dtSCT. DsSCD are *bona fide* empty after purification, highly peptide-receptive and show a remarkable thermal stability that in part could involve the covalently tethered β_2_m molecule.

### 3.4 Development of a dsSCD-based peptide binding assay and application for tumor patient epitope discovery

**Figure 6:**
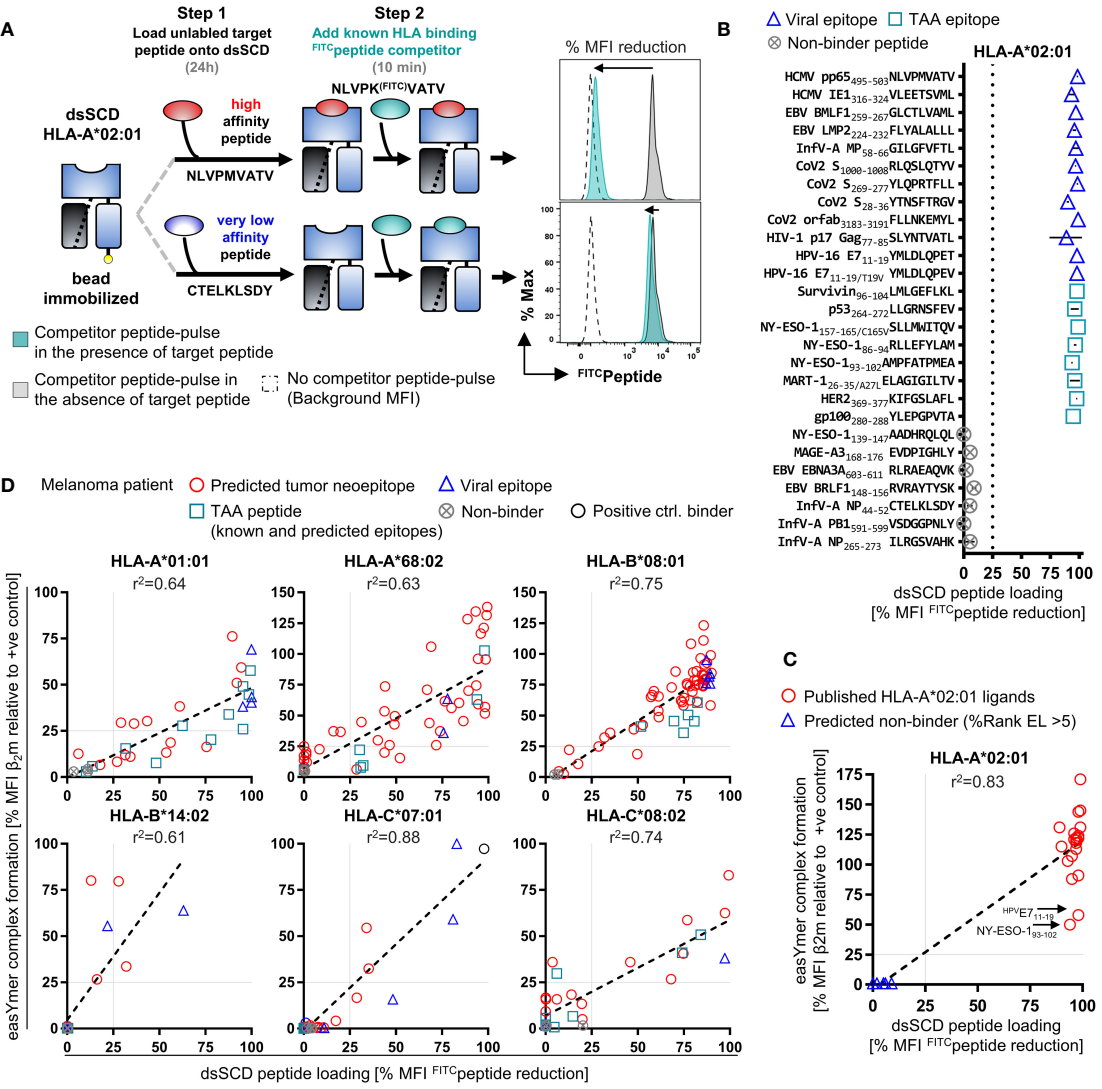
High-throughput dsSCD-based screening assay of peptide binding to HLA-I for *in silico* tumor neoepitope prediction validation. **(A)** Principle of a fast, flow cytometry-based assay for the reliable interrogation of large unlabeled peptide libraries for dsSCD binding exemplarily shown for HLA-A*02:01. As a first step, bead-immobilized dsSCD are loaded overnight with an unlabeled peptide at 10 µM followed by a 10 min pulse with a known FITC-labeled competitor peptide at 1 µM concentration. dsSCD occupancy by an unlabeled high-affinity target peptide, or high dsSCD receptivity to the competitor peptide due to prior loading with a very low-affinity peptide, is indicated by a low ^FITC^peptide signal (i.e. major reduction *vs.* +ve control signal) or a high ^FITC^peptide signal (i.e. minor reduction *vs.* +ve control signal) on the dsSCD-loaded bead, respectively. **(B)** Binding analysis of a panel of 20 known viral and tumor-associated (TAA) HLA-A*02:01 ligands and 7 predicted HLA-A*02:01 non-binding peptides by the dsSCD-based peptide screening assay. The MFI signal reduction in [%] relative to dsSCD-beads that have been loaded with ^FITC^peptide in the absence of a target peptide is shown. 100% MFI reduction indicates an approximation of the complete dsSCD occupancy by a target peptide. **(C)** Comparative peptide binding analysis by HLA-A*02:01 dsSCD peptide binding and easYmer complex formation (ECF) assay using known HLA-A*02:01 ligands. Shown is the dsSCD-bead MFI ^FITC^peptide signal reduction in [%] from **Figure 6B** in correlation with the bead-immobilized HLA-A*02:01 ECF displayed as MFI value of β_2_M in [%] of an ECF performed in the presence of a target peptide relative to an ECF using a designated positive (+ve) control peptide. **(D)** Comparative binding interrogation by ECF and dsSCD-binding assay covering all six HLA-I allotypes of the melanoma patient for *in silico* predicted tumor neoepitopes based on whole-exome sequencing and RNA-Seq data sets of the melanoma patient’s lymph node metastasis as well as selected TAA and viral epitopes. Individual peptides analyzed and peptide dsSCD-binding assay data are listed in **Supplementary Table 3** and **Supplementary Figure 3A**, respectively. **(C, D)** Peptides that display a relative MFI lower than 25% (gray dotted lines) have been empirically determined as poor/non-binders. In all assays shown, the easYmers HLA-B*14:01 and HLA-C*05:01 were used as a surrogate for the patient’s HLA-B*14:02 and HLA-C*08:02 allotypes, respectively, displaying highly similar peptide binding motifs.

We developed a novel dsSCD-based peptide binding assay using high affinity Lys^FITC^-substituted competitor peptides for individual dsSCD HLA-A,B,C allotypes (see **Supplementary Table 1**) based on experimental parameters delineated in **Figure 1** and optimization of incubation time and concentration of the competitor peptide (data not shown). In the binding assay, bead-immobilized dsSCD were pulsed with unlabeled peptides at 10 µM concentration for 18 h before addition of competitor peptide at 1 µM for 10 min, washing and analyzing the bead-associated fluorescence by flow cytometry. As depicted in the schematic drawing of **Figure 6A**, a pre-bound high-affinity peptide will prevent the binding of the FITC-labeled competitor and lead to a strong reduction of the maximal achievable fluorescence intensity, while a pre-bound peptide of very low affinity and thus considered as non-binder will be almost completely replaced by the competitor peptide resulting in a strong FITC signal. To validate the binding competition assay, a set of 12 HLA-A*02:01 peptide ligands derived from viral proteins and 8 known peptide ligands from tumor-associated antigens as well as 7 non-A*02:01 binders was then tested with pp65 N9V^FITC^ competitor peptide (**Figure 6B**, see **Supplementary Table 3** for predicted %Rank EL values). HLA-A*02:01 binders produced 85-100% reduction of the median fluorescence intensity (MFI) by directly loaded N9V^FITC^ and thus confirmed the informative value of the assay. In **Figure 6C** we show a comparative analysis of 20 published viral HLA-A*02:01 ligands and 7 non-binders using the dsSCD-based competition assay and the commercial easYmer-based peptide–β_2_m–HLA heavy chain complex stabilization assay that is read out by binding of an anti-β_2_m antibody. All binders (red circles) cluster with a few outliers around the diagonal at 95-100% N9V^FITC^ MFI reduction while non-binders (blue triangles) cluster around 0-5% MFI reduction, yielding a correlation coefficient of 0.83. Interestingly, two binders, HPV E7_11-19_ and NY-ESO-1_93-102_ relatively weakly stabilized HLA-A*02:01 easYmers whereas the dsSCD competition assay indicated strong binding.

Next, we set out to apply the MediMer peptide binding assay to the six HLA-A,B,C allotypes and 166 unique tumor neoepitope peptides predicted by NetMHCpan 4.1 (61) after whole exome sequencing of tumor cells from a lymph node metastasis of an advanced-stage melanoma patient (**Supplementary Table 3**). Individual binding data of predicted tumor neoepitope peptides to HLA-A*01:01, A*68:02, B*08:01, B*14:02, C*07:01 and C*08:02 dsSCD, respectively, is shown in **Supplementary Figure 3A**. In this figure we also show dsSCD binding values for several published allotype-specific peptide ligands derived from non-mutated tumor-associated antigens (TAA) and from viral proteins (see **Supplementary Table 3** for details of peptides and predicted binding values). For the same panel of peptides we conducted easYmer complex formation assays in parallel. In lack of an HLA-C*08:02 easYmer, the HLA-C*05:01 easYmer was used instead because it has a very similar peptide binding motif, and the HLA-B*14:01 easYmer was used as a surrogate for the patient’s B*14:02 allotype. Binding values in dsSCD and easYmer assays were relatively consistent with correlation coefficients of 0.61-0.88 across all allotypes studied (**Figure 6D**). We conclude that, although the novel MediMer peptide binding competition assay and the easYmer pMHC-I complex formation assay are obviously different in biochemical terms, results seem to be sufficiently consistent to be combined if required by the availability of reagents.

### 3.5 dsSCD multimer-based screening for T cells recognizing tumor neoepitopes

**Figure 7:**
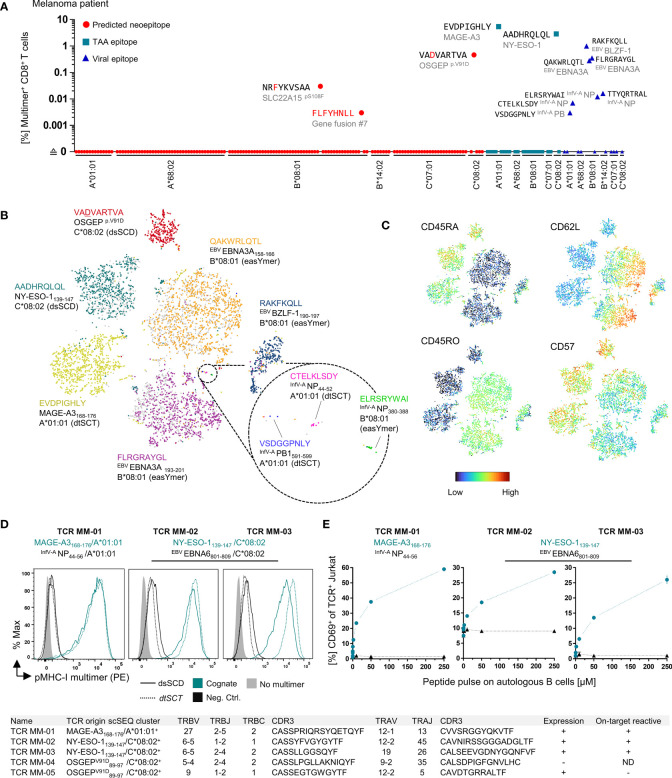
Detection of neoepitope- and TAA-specific CD8^+^ T cell populations in the peripheral blood of melanoma patient using multimerized easYmers, dtSCTs and dsSCD (MediMers) in a complementary manner. **(A)** Identified antigen-specific CD8^+^ T cell populations in the patient’s peripheral blood by dual color-encoded pMHC-I multimer staining covering all six HLA alleles of a melanoma patient. In total, 148 pMHC-I multimers were generated (107 predicted neoepitopes, 24 non-mutant tumor-associated antigens (TAA) and 17 viral epitopes) on the basis of a dsSCD for HLA-C*08:02 and easYmers covering the remaining five HLA-I allotypes. EasYmer HLA-B*14:01 was used as a surrogate for the patient’s HLA-B*14:02 allotype. For the initial cell staining up to 60 dual color-encoded pMHC-I multimer pairs were used in one single pMHC-I library and detected pMHC-I multimer^+^ populations were verified afterwards by at least two additional independent stainings with up to 10 dual color-encoded pMHC-I multimer pairs. Representative dot plots are shown in **Supplementary Figure 3D**. Zero values were converted to 0.0001 to allow for plotting on a log scale. **(B, C)** pMHC-I multimer-guided single-cell TCR repertoire and cell surface protein expression analysis. pMHC-I multimer^+^ CD8^+^ T cells were cell sorted using a pMHC-I multimer library comprising uniquely DNA-barcoded and population size-dependent dual color-encoded pMHC-I multimer pairs (see **Supplementary Figure 2C**) combined with a panel of 30 DNA-barcoded cell phenotyping antibodies (**Supplementary Table 4**). Multimerized dsSCD, easYmer and dtSCT were used in combination. An experimentally obtained 10x scSeq data set is shown as tSNE plot based on surface marker expression including the pMHC-I multimer labeling of a total of 3236 individual cells represented by dots. Cell clustering based on pMHC-I multimer barcode detection **(B)** and cell surface expression of selected T cell memory markers **(C)** is shown. **(D, E)** Validation of cloned MAGE-A3 and NY-ESO-1-specific TCR from the 10x scSEQ data set. TRBV/TRBJ and TRAV/TRAJ subtypes and respective CDR3 sequences are displayed in the table at the bottom. **(D)** Antigen-specific staining of a MAGE-A3_168-176_/A*01:01-specific TCR (MM-01) and two NY-ESO-1_139-147_/HLA-C*08:02-specific TCR (MM-02 and MM-03) expressed in J76^CD8+^ cells with multimerized dtSCT and dsSCD representing the cognate (teal lines) or a viral control (black lines) epitope. **(E)** Co-culture of TCR-expressing J76^CD8+^ cells with autologous peptide-pulsed B cells. The expression of early T cell activation marker CD69 ± SD in [%] of TCR^+^ J76^CD8+^ cells after 18h co-culture in triplicates in presence of cognate (teal) or a control (black) peptide at various concentrations. Two cloned dominant TCRs (MM-04 and MM-05) derived from the OSGEP^V91D^/HLA-C*08:02 multimer scSEQ cluster lacked expression in J76^CD8+^ cells or were not stained by corresponding the pMHC-I multimer, respectively. ND, not determined.

Using the peptide binding data obtained in the MediMer screening of the melanoma patient we endeavored to identify neoepitope-reactive T cell populations in the peripheral blood of the melanoma patient. pMHC-I multimers were assembled for each of the 107 selected neoepitopes accompanied by screening for 24 epitopes derived from tumor-associated antigens and 17 epitopes from viral peptides predicted to be presented by any of the patient’s six HLA-A,B,C allotypes (**Figure 7A**). By staining CD8^+^ T cells from PBMC using multimerized easYmers and dsSCD in a complementary manner that were color-coded with streptavidin-fluorochrome conjugates in a 60-fold matrix, we detected 3 HLA-B*08:01- and HLA-C*08:02-restricted T cell populations reactive with tumor-specific point mutations and a neo-sequence resulting from an out-of-frame gene fusion event. In addition, MAGE-A3/A*01:01 and NY-ESO-1/C*08:02 antigen-reactive T cell specificities were detected as well as seven T cell populations specific for known EBV- and influenzavirus-derived epitopes that we had selected for the six HLA allotypes. Exemplarily, color-coded 10-plex multimer stainings are presented in **Supplementary Figure 3B**, demonstrating the existence of CD8^+^ T cells subpopulations recognizing the tumor neoepitopes, OSGEP_V91D_/HLA-C*08:02 (0.46%), SLC22A15_S108F_/HLA-B*08:01 (0.028%) and fusion #7 neo-sequence/HLA-B*08:01 (0.002%). For the two identified neo-epitopes based on single nucleotide variations we conducted peptide binding assays including the corresponding wild-type peptides (**Supplementary Figure 3C**). Interestingly, in both the OSGEP_V91D_ (VA**D**VARTVA) and the SLC22A15_S108F_ (NR**F**YKVSAA) neoepitope peptides the mutation created a *de novo* anchor residue enabling binding to HLA-C*08:02 and HLA-B*08:01, respectively, while wild-type variants did not bind.

To identify sequences of TCRs recognizing neo-epitopes by single RNA cell sequencing, epitopes from shared tumor-associated antigens as well as viral epitopes, CD8^+^ T cells from the peripheral blood of the melanoma patient were labeled with pools of peptide-loaded MHC-I molecules multimerized with DNA-barcoded PE-conjugated dCODE Dextramer^®^ reagents (**Figure 7B**, **Supplementary Figure 2C**, **Supplementary Table 4**). Relying on equal labeling performances of the three types of multimers (see **Figure 3D**), we combined the multimer labeling of the patient’s T cells, peptide-loaded dsSCD, peptide-loaded easYmers and dtSCTs due to varying availabilities of the multimer reagents at the time of the experiment (**Figure 7B**). To increase the likelihood to detect small subpopulations, dual color-coded T cell populations occurring at high (>1%, PE^+^ BUV395^+^), intermediate (~0.1%-1%, PE^+^ BV421^+^) and low (~0.005-0.1% PE^+^ APC^+^) frequency were separately FACS-sorted and mixed in adjusted numbers under-representing T cell specificities of high and intermediate frequencies (**Supplementary Figure 2C**). A pool of 30 antibodies reacting with T cell lineage and differentiation markers and labeled with Total SeqC^®^ DNA feature barcodes were used simultaneously in order to phenotype antigen-specific T cells on the single cell level. After scRNA-Seq of pMHC-I multimer^+^ T cells accomplished by the 10x Genomics platform, we obtained 180 TCRα/β clonotypes, antibody-based phenotypes and gene expression data from 3236 barcoded cells that are depicted in the feature barcode-based t-SNE plot of **Figure 7B**. T cell specificities defined by respective pMHC-multimers are shown in differently colored t-SNE clusters of greatly varying sizes. Unfortunately, we were unable to retrieve SLC22A15_S108F_/B*08:01 and Fusion#7/B*08:01 labeled populations after scRNA-Seq, which was likely due to insufficient B*08:01 easYmer formation and a too low cell population frequency, respectively, for those two epitopes. Surface marker expression analysis of the identified pMHC-I multimer^+^ populations clearly characterized the NY-ESO-1_139-147_, MAGE-A3_168-176_ and OSGEP_89-97/V91D_ reactive populations as belonging to the CD57^+^CD45RA^+^ exhausted effector memory T_EMRA_ subset, while EBNA3A_158-166_ and EBNA3A_193-201_ clustered to both the CD45RO^+^/CD62L^+^ central and CD45RO^+^/CD62L^–^ effector memory (T_CM_/T_EM_) subsets (**Figure 7C**).

One MAGE-A3_168-176_/A*01:01 and two NY-ESO-1_139-147_/C*08:02-reactive TCR, the latter being of a so far unreported specificity, were cloned, expressed in Jurkat 76/CD8 cells and validated by successful staining with dsSCD and dtSCT loaded with the respective peptides (**Figure 7D**). These TCR-transfected Jurkat 76/CD8 cells were also activated by peptide-pulsed autologous CD40L-expanded B cells showing different sensitivities with regard to CD69 early activation marker upregulation in a peptide titration (**Figure 7E**). Two putative OSGEP_89-97/V91D_/C*08:02 reactive TCR were cloned, however, unfortunately either failed to be expressible or could not be labeled by cognate pMHC-I multimer after expression in Jurkat 76/CD8.

To further substantiate the usefulness of the MediMer platform for the screening of peptide binding to various HLA-C allotypes, we interrogated the NY-ESO-1 epitopes 139-147 (AADHRQLQL) and 96-104 (FATPMEAEL) in a binding assay with a library of 14 HLA-C dsSCD allotypes (**Supplementary Figure 3D**). Clearly, the binding NY-ESO-1_139-147_ was restricted to HLA-C*05:01, C*08:02 and C*15:02, while NY-ESO-1_96-104_ binding was more promiscuous among the tested HLA-C allotypes.

Taken together, dsSCD can be manufactured matching all six allotypic HLA-A,B,C molecules of an individual tumor patient, can be used as a high-throughput screening platform for allotype-specific peptide ligand validation and for pMHC-I multimer-guided scRNA-Seq for comprehensive TCR discovery combined with immune cell phenotyping.

## 4 Discussion

In the emerging field that is dedicated to the discovery of T cells with specificity for tumor-associated peptide antigens there is a high need for specific and highly sensitive tools capable of high throughput screening in order to detect rare T cell populations in the peripheral blood and tumor infiltrate (63, 64). The MHC-I multimer technology has overcome the problem of low affinity interactions between monomeric MHC-I molecules and antigen-specific T cell receptors that are typically 3–4 orders of magnitude lower than antibody–protein antigen interactions (65). Multimerization of biotinylated peptide-loaded MHC-I heavy chain/β_2_-microglobulin complexes on fluorochrome-conjugated streptavidin has been shown to sufficiently improve binding avidities to enable flow-cytometric detection of various T cell populations of greatly varying frequencies and has been ever since a valuable assay tool that is subjected to a constant evolution (66–68). Furthermore, the simultaneous usage of multiple fluorochrome-streptavidin variants complexed with individual pMHC-I complexes facilitates in one staining a high-dimensional multiplex analysis (2, 3). Moreover, the MHC-I multimer technology has been combined with DNA barcoding and subsequent pMHC-multimer-guided single-cell TCR sequencing and in-depth gene signature analysis of antigen-specific populations as shown recently (69).

As a major advantage, pMHC multimer stainings can be applied independently of the naïve, memory or exhausted functional status of the analyzed T cell population and no preparation of antigen-presenting cells is required. Furthermore, pMHC multimer stainings often show higher sensitivities compared to most other functional assays, such as ELISpot and intracellular cytokine staining (70). This is important since typically tumor-neoantigen-specific T cells occur in *ex vivo* peripheral blood lymphocytes at frequencies of only 0.02% (1 in 5000 T cells) to 0.0007% (1 in about 150,000 T cells) and lower (71, 72) making it often a necessity to perform *in vitro* stimulations with peptide pools combined with pMHC-I multimer stainings in order to detect neoantigen-specific T cell populations in peripheral blood on a regular basis (73).

A limitation of the pMHC multimer technology is that the MHC allotypes of the patient need to be known and the many potential antigenic epitopes need to be accurately predicted to limit pMHC multimers libraries to reasonable workloads and at the same time also match the often limited availability of tumor patients’ blood samples and the urgent medical need.

To manufacture soluble MHC-I molecules, recombinant MHC-I heavy chain molecules lacking transmembrane and intracytoplasmic domains and recombinant β_2_m were conventionally produced in *E. coli* by a laborious procedure (74). Inclusion bodies containing expressed proteins need to be harvested, washed and solubilized in urea-containing buffer. Refolding of the denatured heavy chain and β_2_m is performed in folding buffers under reducing conditions at pH 8 for several days in the presence of final peptide ligands for the production of a particular peptide-loaded MHC-I monomer (1), or in the presence of UV-cleavable conditional peptide ligands, for the purpose of generating pMHC-I multimer libraries by peptide exchange (3, 4, 10, 14, 75–77). After refolding, pMHC-I complexes are usually enzymatically biotinylated by BirA biotin ligase at a biotin acceptor tag sequence (AviTag™) at the C-terminus of the heavy chain, followed by purification through size exclusion chromatography.

Although a larger panel of 26 HLA-A,B,C allotypes endowed with conditional ligands have been reported (14, 76, 78, 79), up to date there is only the limited number of 8 HLA-A,B,C UV-exchangeable allotypes commercially available as Flex-T™ reagents (BioLegend). This small selection does not satisfy the needs of immune oncologists who intend to screen neoepitope-specific T cells reactive against the entirety of HLA allotypes of all patients.

Other experimental methods described for only a small collection of recombinant MHC-I molecules (HLA-A*02:01, H-2K^b^) utilized temperature-dependent peptide exchange of low-affinity placeholder peptides in conventionally refolded, recombinant bacterial MHC-I proteins (13, 80) or tapasin-mediated exchange of pMHC-I (HLA-A*02:01, H-2L^d^, H-2D^d^) refolded in the presence of truncated low affinity peptides (81).

Recently, disulfide-stabilized MHC-I heavy chains have been used for refolding with β_2_m and a stabilizing dipeptide (10). After purification, these molecules are empty and highly receptive for conventional MHC-I peptide ligands, and thus represent ideal tools for the generation of pMHC-I libraries. Peptide-receptive empty MHC-I molecules appear to be superior to systems utilizing peptide exchange of soluble heterotrimers preloaded with endogenous ligands (82) because the efficiencies of peptide exchange by new ligands of low and intermediate affinity is unpredictable. However, so far only a small number of disulfide-stabilized allotypes are commercially available which might be due to technical difficulties associated with the refolding of various disulfide-stabilized HLA-A,B,C heavy chains with β_2_m that, except for HLA-A*02:01, A*24:02 and H-2K^b^ molecules that refold in the presence of dipeptides, required the use of UV-cleavable placeholder peptides during *in vitro* refolding (77).

A successful alternative strategy employs pre-oxidized, bacterially produced heavy chains that can be efficiently biotinylated by BirA overexpressed in *E. coli* co-transformed with the MHC-I plasmid (7, 83). Resulting oxidized, denatured heavy chain can be easily refolded with an excess of β_2_m and suitable peptide and form tetramers with fluorochrome-labeled streptavidin in one-pot reactions at miniature scale. While the purification of correctly oxidized MHC-I isoforms appears to be demanding (84), the downstream application is easy, flexible and capable of high-throughput screening of antigen-specific T cells. A large number of HLA-A [25], HLA-B [45] and HLA-C [12] allotypes are presently available as peptide-refoldable easYmers^®^ for flow cytometry (immunAware/Immudex). Due to their good performance, in this work easYmers have been used as benchmark for our own dsSCD. Notably, however, until recently only ca. 80% of the MHC-I allotypes expressed by 23 tumor patients included in neoepitope screening campaigns in our laboratory were covered by the easYmer platform. Moreover, the routine use of commercially available pMHC-I multimer platforms is fraught with a considerable financial burden.

With the development of the herein reported MediMer platform utilizing disulfide-stabilized peptide-receptive recombinant MHC-I molecules produced in mammalian cells we fill the gap of patient-tailored production of recombinant rare HLA-A,B,C allotypes. As we demonstrate in the present work, these can be used in TCR discovery pipelines including a bead-based screening assay for libraries of individual putative neoepitope peptide ligands and the formation of sensitive dsSCD-based pMHC-I multimers usable for the *ex vivo* staining of antigen-specific T cells in peripheral blood, for the isolation of multimer-binding T cells by flow cytometry or magnetic beads, and finally for the validation of cloned TCR expressed as transgenes in T cells.

Except for the cloning of new HLA-A,B,C allotypes harboring the Y84C and A139C mutations that form the stabilizing disulfide, little hands-on time is required after purification via a C-terminal histidine tag. Due to their metabolic biotinylation by co-expressed BirA ligase, peptide-receptive dsSCD molecules are ready to be loaded to streptavidin-coated beads or to be incorporated into pMHC-I multimers based on streptavidin molecules conjugated to a large variety of fluorophores enabling combinatorial color coding. As an obvious advantage, the production of SCT in mammalian producer cells (25, 29, 30, 85, 86) and with this work also of dsSCD is circumventing the limitations of the technically demanding *in vitro* refolding of proteins obtained from *E. coli* inclusion bodies requiring the skills of specialized biochemists. We demonstrate superior thermal stability of empty dsSCD that can be stored frozen as well as peptide-loaded at 4°C and thus represent a versatile off-the-shelf tool.

The principle of tethering β_2_m via flexible linker to the C-terminus of soluble mouse MHC-I heavy chains to render peptide-receptive single-chain MHC-I molecules has initially been described about thirty years ago (87). Toshitani and colleagues first reported a membrane-bound HLA-A2 single-chain dimer with N-terminally tethered β_2_m (88). A recent study employed His-tagged, affinity-maturated HLA-K^b^ chimeric ectodomains in peptide exchange assays for the mapping tumor-associated epitopes (82). In another study, soluble single-chain dimers of β_2_m and various wild-type HLA-A,B,C allotypes were employed for a detailed mass spectrometric analysis of HLA peptide ligands (89). Both groups used HEK293 as producer cells. For still unclear reasons we failed to produce Fc-tagged single-chain dimers in HEK293 cells, neither as wild-type HLA-A*02:01 SCD-Fc (data not shown), nor as SCD-Fc containing the groove-opening mutation Y84A (21) (data not shown) nor as dsSCD-Fc (see **Figure 1**). Since respective molecules were detected by an intracellular staining using BB7.2, an antibody recognizing folded HLA-A2 molecules (**Figure 1B**), as well as by anti-mouse IgG, we suppose that metabolically biotinylated dsSCD were retained by ER quality control mechanisms that were apparently not active in CHO-S cells since the latter secreted significant amounts of dsSCD-Fc (see **Figure 1D**). It seems possible that a partial or complete inability of soluble dsSCD to interact with the TAP-tapasin loading peptide complex, due to the mutation of the tapasin-interacting Tyr84 residue (90), contributed to the intracellular retention of dsSCD-Fc in HEK293 cells as the lack of peptide cargo might have been sensed by ER or cis-Golgi quality control mechanisms of HEK293 cells. On the other hand, H-2K^b^ molecules harboring the same Y84C/A139C disulfide bridge were reported to overcome intracellular retention in peptide loading complex-deficient mouse fibroblasts (9). Alternatively, the production of dsSCD in CHO-S cells at 32°C might have facilitated escape from quality control and subsequent secretion, in line with older reports showing that in peptide transporter-deficient cells, membrane-bound peptide-free MHC-I molecules are efficiently cell surface-expressed at reduced temperatures of 19-33°C (91, 92). Nevertheless, the observation that no endogenous peptide ligands could be detected by mass spectrometry in CHO-S-produced HLA-A*02:01 dsSCD (see **Figure 1G**) is in our opinion of great practical value since empty dsSCD should be more easily loadable with peptides of low and intermediate affinity thereby expanding the screening space for potential neoepitope ligands.

As previously reported for membrane-bound H-2K^b^ molecules harboring the peptide binding groove-stabilizing Cys84-Ala139 disulfide bond, the affinity of disulfide-stabilized heavy chains for β_2_m is remarkably increased (9). Hence is not surprising that cleavage of the flexible linker between β_2_m and heavy chain using an artificially introduced HRV 3C site had no major influence on the peptide receptivity of dsSCD (see **Figure 5**). Nevertheless, we assume that the presence of tethered β_2_m in single-chain dimers is of benefit for the long-term stability of empty dsSCD *in vitro*, as in contrast to non-covalent complexes of free heavy chain and β_2_m, tethered β_2_m can be conceived to quickly reassemble after occasional partial or complete dissociation, thereby impeding denaturation of the empty heavy chain. We have shown that the time- and temperature-dependent deterioration of the peptide binding capacity of dsSCD is almost completely prevented in the continuous presence of an excess of exogenous peptide even at elevated temperature (see **Figure 4C**), reaffirming the known importance of peptide for the stability of the heterotrimeric complex (91, 92).

Using empty dsSCD as peptide receptors, the determination of K_D_ dissociation constants is straightforward for peptides harboring fluorochrome-tagged amino acids such as lysine^FITC^ by extrapolating saturation binding in titration curved (see **Figure 1E**; **Supplementary Figure 1B**, while analysis of binding affinities of unlabeled peptides does not appear trivial. A previous study employing preincubation of unlabeled ligand for periods up to 24 h followed by competition with radioactively labeled competitor ligand for 15-30 min (93), suggests that the ‘preincubation endpoint approach’ resembling the conditions of the dsSCD competiton assay used herein, could enable the estimation of K_D_ values of unlabeled peptides by simply determining their IC_50_ values for inhibition of binding of FITC-labeled index peptides to a given dsSCD.

By co-expression of an ER-targeted *E. coli* BirA biotin ligase possessing a C-terminal ER retention signal (62), we facilitate metabolic biotinylation of the soluble ds-SCD molecules tagged with BirA ligase recognition sequence (AviTag) circumventing the need for enzymatic biotinylation of purified pMHC-I molecules (86). While metabolic biotinylation appeared to be sufficient to induce a streptavidin-mediated gel shift of a major proportion of all tested HLA-A,B,C allotype monomers and for the loading of streptavidin beads as well as for the formation of streptavidin-based pMHC-I multimers conducted in this work, metabolic biotinylation in CHO-S cells was not as efficient as metabolic biotinylation in BirA-overexpressing *E. coli* for which biotinylation efficiencies of 85-100% were reported for >40 HLA-A and HLA-B alleles (7), or as metabolic biotinylation of dtSCT in 293-F cells conducted in this study. It is possible that insufficient quantities of ER-retained BirA ligase were co-translationally introduced into ER subcompartments where nascent dsSCD molecules were inserted, or that the kinetics of ER-translocated BirA ligase was too slow to completely biotinylate all dsSCD molecules probably leaving the ER and ER-recycling compartments rapidly. In future experiments it will be attempted to improve metabolic biotinylation by generating a CHO-S producer line stably transfected with ER-resident BirA ligase and analyze the option of co-translational biotinylation of nascent dsSCD molecules by over-expression of cytoplasmic BirA ligase.

As shown in this work the newly established MediMer peptide binding assay that is based on equilibrium phase loading with unlabeled test peptides followed by a flow cytometry read-out using a fluorescently labeled competitor peptide, is capable to be conducted in a high-throughput fashion similar to the easYmer complex formation assay that measures the stable association of free β_2_m to free biotinylated heavy chains in the presence of stabilizing peptides. These assays are based on different biochemical principles and it is therefore not surprising that we observed minor differences in the binding values obtained for individual members of large panels of peptides, that were only *in silico* predicted to be ligands for an HLA-A,B,C allotype of choice but had not been validated before. Since the overall performance of the two binding assays appear to be sufficiently consistent, they could be used in a combinatorial manner to arrive at a selection of peptides that is subsequently used for pMHC-I multimer formation with the goal of identifying respective CD8^+^ T cell populations in peripheral blood or tumor infiltrates directly *ex vivo*, or after *in vitro* peptide restimulations.

Undoubtedly, peptide-loaded dsSCD representing various MHC-I allotypes performed equally well in pMHC-I multimers as compared to pMHC-I multimers formed with easYmers or disulfide-trapped single-chain trimers with regard to the simultaneous discrimination of T cell populations of greatly different frequencies by combinatorial color coding (see **Figure 3**). We conclude from the very slow peptide dissociation of peptide-loaded dsSCD at 4°C and ambient temperature (see **Figure 4**), that peptide-dsSCD complexes appear to be highly stable once formed, and due to the high specificity of T cell labeling, suited to be used for the search for very small T cell population below 0.1% frequency that are often encountered when analyzing T cell specificities directly *ex vivo*.

Up to the present time we have been able to successfully produce by transient gene expression in CHO-S producer cells more than 10 HLA-A, 8 HLA-B and 14 HLA-C metabolically biotinylated dsSCD allotypes (**Figure 2**, **Supplementary Figure 1** and data not shown). This success prompted us to screen T cells from an HLA-C*08:02-expressing melanoma patient for tumor-antigen reactive T cells with the herein reported HLA-C*08:02 dsSCD that is not available as easYmer. We first produced 6 dsSCDs matching the HLA-A,B,C allotypes of that patient and employed them in the newly developed MediMer peptide binding assay to screen a large panel of potential neoepitope peptides, peptides derived from shared tumor-associated antigens as well as known viral epitopes for *in vitro* binding to dsSCD and easYmers (see **Figure 6D**). This screening campaign revealed a large number of confirmed binders that were subsequently incorporated in individual dual color-coded pMHC-I multimers and used in multiplex labeling reactions of peripheral blood CD8^+^ T cells from the patient (**Figure 7A**). Using HLA-C*08:02 dsSCD we detected two T cell populations recognizing tumor-derived peptides, one directed against a tumor neoepitope and the other specific for a novel NY-ESO-1 epitope. Using peptide-loaded dsSCD and dtSCT representing A*01:01, B*08:01, and B*14:01 allotypes, we were able to label small neo-epitope specific T cell populations and larger populations reactive with epitopes derived from viral recall antigens that are expected to exist in greater frequencies (**Supplementary Figure 3**). Enabled by DNA-barcoded pMHC-I multimers representing a combination of peptide-loaded dsSCDs, easYmers and peptide-tethered dtSCTs, we successfully conducted scRNA-Seq of the melanoma patient-derived pMHC-I multimer^+^ CD8^+^ T cell population leading to the discovery of novel MAGE-A1/A*01:01 and NY-ESO-1/C*08:02 T cell receptors that could be functionally validated by recombinant expression in Jurkat reporter cells and that could be useful for other patients sharing these HLA-I alleles.

Taken together, we herein presented that the technically easily accessible MediMer platform of peptide-receptive disulfide-stabilized single-chain dimers can be rapidly deployed to screen a large panel of potential tumor-associated peptides in a binding assay, facilitate the detection of antigen-specific T cell populations even at very low frequencies in the peripheral blood of patients and enables pMHC-I multimer-guided scRNA-Seq for the identification and cloning of respective TCR receptors that can be potentially used in adoptive transfer regimens with TCR-transgenic autologous T cells.

## Data availability statement

The whole-genome sequencing, RNA sequencing and single-cell RNA sequencing datasets presented in the study are deposited in the European Genome Archive (EGA) repository, accession number EGAS50000000065. The study contains the sequencing datasets EGAD50000000092 (DAC: Momburg/Meyer) and EGAD50000000093 (DAC: HIPO DACO). Additional supporting data are available from the corresponding authors upon reasonable request.

## Ethics statement

Blood samples from healthy donors were collected according to the principles of the Declaration of Helsinki and were obtained from the Deutsches Rotes Kreuz (DRK) Blutspendedienst Baden-Württemberg-Hessen gGmbH, Mannheim, Germany. All experiments with patient-derived material were conducted in accordance with the Declaration of Helsinki and a written informed consent was obtained from the patient, approved by ethics votes S-022/2013, S-206/2011 (MASTER trial) and S-207/2005 (NCT Biobank), renewed on 10 September 2018, Ethics Committee of the Medical Faculty of Heidelberg University, Heidelberg, Germany.

## Author contributions

Conceptualization: MM, FM; Methodology: MM, CP, TB, JB, SB, DI; Software: PC, YL; Validation: MM, CP, TB, JB, PC; Formal analysis: MM, CP, TB, JB, PC, YL, AR, MR, IZ, FM. Investigation: MM, CP, TB, JB, SB, NB, CT, LW, RP, DI, PaS, KL, AR, JH, MR IZ, FM. Resources: DI, PaS, SE, PeS, IP, MP, SF, AR, JH, DJ, IZ, FM; Data curation: MM, PC; Writing – original draft: MM, FM; Writing – review and editing: All authors; Visualization: MM, CP, TB, JB, FM; Supervision: MM, FM; Project administration: MR, IZ; Funding acquisition: MM, SBE, MP, IP, SF, AR, IZ, DJ, FM. All authors contributed to the article and approved the submitted version.

## Supplementary material

Supplementary Figure 1 Production of various dsSCD allotypes and positional analysis of lysine^FITC^ substituted peptides. **(A)** Exemplary SDS-PAGE analysis of affinity chromatography-purified dsSCD produced as Fc-free variants in CHO cells. dsSCD biotinylation was confirmed by gel shift upon equimolar addition (+) of streptavidin prior to the gel electrophoresis. **(B)** Systematic binding analysis of FITC-lysine (K^FITC^)-substituted NLVPMVATV peptides towards bead-immobilized HLA-A*02:01 dsSCD. Beads were pulsed with the indicated concentrations of ^FITC^peptides for 18 hours. Non-linear regression (one-site specific binding) of the FITC MFI against the peptide concentration and calculated K_D_ values in µM are shown. For comparison, the NetMHCpan 4.1-based binding motif of naturally bound, eluted ligands (EL) of HLA-A*02:01 is shown above the lysine-substituted NLVPMVATV sequence (https://services.healthtech.dtu.dk/services/NetMHCpan-4.1/). HLA-A*02:01 %Rank EL prediction values are shown for the indicated peptides with K substitution and their classified binding levels as putative strong binders (SB, %Rank EL <0.5), weak binders (WB, %Rank EL <2) and non-binders (nonB, %Rank EL >2). The FITC conjugation is neglected by this prediction.

Supplementary Figure 2 Flow cytometry gating strategies. **(A)** Antigen-specific TCR validation of stably transfected Jurkat 76 CD8^+^ (J76^CD8+^) exemplary shown for the NY-ESO-1_139-147_/HLA-C*08:02-specific TCR MM-03 (see **Figure 7**). pMHC-I multimer binding (left histograms) or CD69 upregulation in the presence of stimulation (right histograms) is always shown for the TCR^+^/CD8^+^ J76^CD8+^ fraction. **(B)** Dual color-encoded pMHC-I multimer analysis of healthy donor (HD-01) and patient-derived CD8^+^ T cells is exemplary shown for one dual-color encoded pMHC-I multimer^+^ population of HD-01. HD-01 CD8^+^ T cells were stained with a pool of three dual-color pMHC-I multimer pairs, each pair associated with a different peptide and a unique dual color combination on the basis of streptavidin-conjugated fluorochromes APC, PE and BV421 used in this experiment for the pMHC-I multimer preparation. For analysis, single, living CD3^+^ CD8^+^ T cells were identified following the definition of positive events in each pMHC-I multimer channel as well as the generation of respective NOT gates thereof. To display a single dual color pMHC-I multimer population using Boolean gating, two positive gates (APC, PE) and here one NOT gate (BV421) are combined as a single AND gate (i.e., APC+ AND PE+ AND BV421–). Finally, the APC+ AND PE+ AND BV421– gate is combined as an OR gate with [APC– AND PE– AND BV421–] as shown for the ^HCMV^pp65/A*02:02-specific CD8^+^ T cell population of HD-01. **(C)** Used gating strategy for cell sorting of dual color and DNA-barcoded (dCODE dextramers^®^) pMHC-I multimer^+^ CD8^+^ T cells of the melanoma patient for subsequent single-cell sequencing (scSEQ). Based on prior dual-color encoded pMHC-I multimer analysis of CD8^+^ T cells *ex vivo* from the patient, all identified pMHC-I multimer^+^ populations were pre-clustered for cell sorting depending on their frequency as low (< 0.1%), intermediate (0.1%–1.0%) or high (>1%) frequent and associated with the fluorochromes APC, BV421 or BUV395, respectively, to ensure that also populations of very low frequency were included in the scSEQ. For the dual-color and DNA-barcoded pMHC-I multimer library generation, each epitope was encoded by a uniquely DNA-barcoded, PE-conjugated dextramer as well as paired with the same pMHC-I multimerized with streptavidin APC, BV421 or BUV395 depending on the frequency (see also **Supplementary Table 4**). For the sorting, single living CD3^+^ CD8^+^ T cells and pMHC-I dextramer-PE^+^ were selected following the separation of the respective dual-color positive pMHC-I multimer^+^ populations. Finally, individual, separated pMHC-I multimer^+^ populations of low, intermediate or high frequency were mixed for the scSEQ in a defined ratio favoring small populations.

Supplementary Figure 3 dsSCD peptide binding validation of *in silico* predicted neoepitopes of a melanoma patient’s lymph node metastasis and identification of three neoepitope-specific and two TAA-specific CD8^+^ T cell populations in autologous peripheral blood. **(A)** HLA binding analysis of *in silico* predicted tumor neoepitopes and selected known and predicted tumor-associated non-mutated epitopes (TAA) using dsSCD-based peptide binding assays covering all six HLA-I allotypes of the patient. Shown is the MFI signal reduction ± SD in [%] relative to dsSCD-beads that have been loaded with ^FITC^peptide in the absence of a target peptide. Individual peptides are listed in top-down order according to their *in silico* binding prediction score (%Rank EL) for the respective HLA allotypes (also see **Supplementary Table 3**). Peptides are considered as HLA binders if they display a relative MFI reduction higher than 25% (dashed horizontal lines). **(B)** Labeling of the melanoma patient’s autologous peripheral CD8^+^ T cells *ex vivo* with dual-color encoded HLA-I multimers generated on the basis of dsSCD, dtSCT or commercial easYmer^®^. Representative dot plot of selected pMHC-I multimer^+^ populations that have been detected in three or more independent experiments are shown. **(C)** Comparative peptide-HLA binding analysis of predicted neoepitopes SLC22A15^p.S108F^ and OSGEP^p.V91D^ and their wild-type counterparts using an HLA-B*08:01 easYmer complex formation assay and HLA-C*08:02 dsSCD peptide binding assay, respectively. **(D)** Binding analysis of the NY-ESO-1_139-147_ (magenta) and NY-ESO-1_96-104_ (black) peptide towards various HLA-C dsSCD allotypes. The experiments shown in **(A, C, D)** were conducted in technical triplicates. SD, Standard deviation of the mean.
