# Supplementary figures and images for: MediMer: a versatile do-it-yourself peptide-receptive MHC class I multimer platform for tumor neoantigen-specific T cell detection
Source: Front Immunol. 2024 Jan 4;14:1294565. doi: 10.3389/fimmu.2023.1294565 (PMC10794645; doi:10.3389/fimmu.2023.1294565)

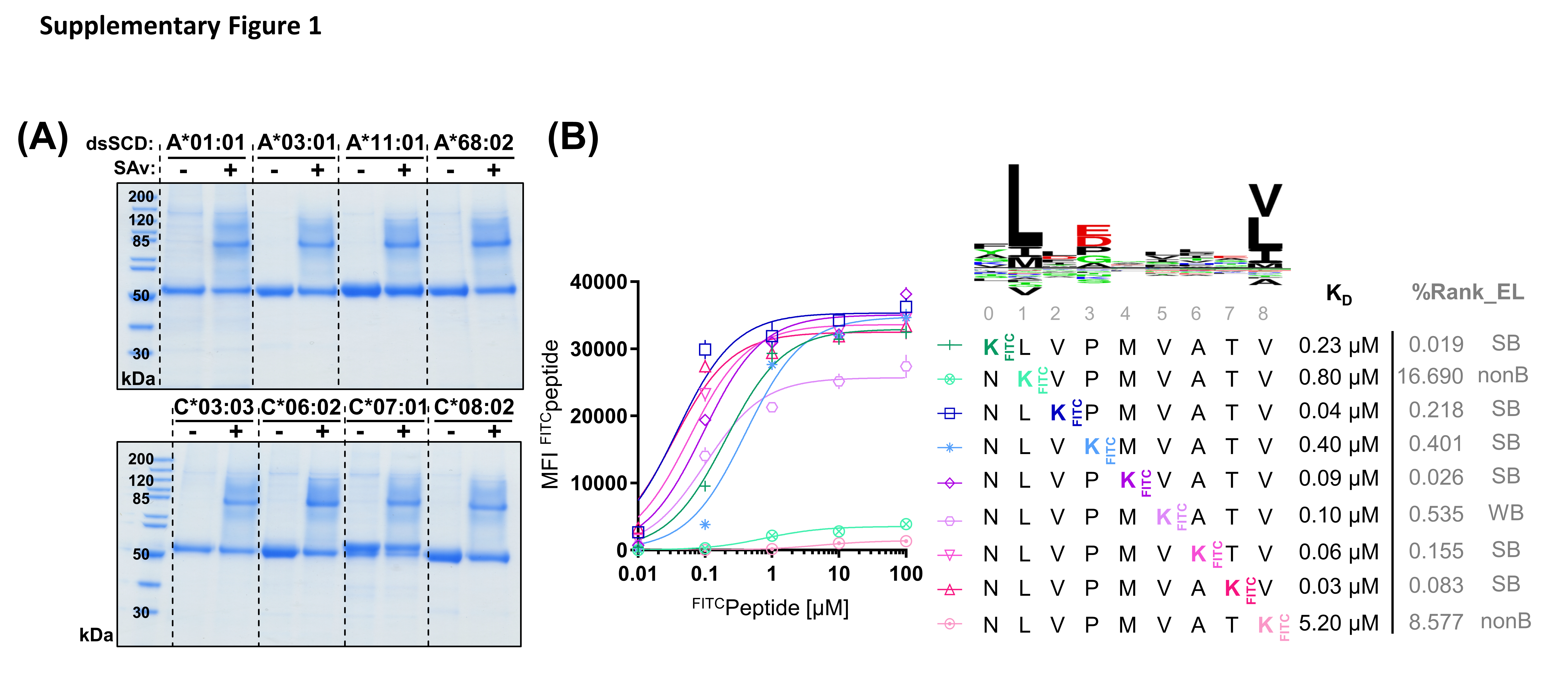

Supplement: Supplementary Figure 1 — Production of various dsSCD allotypes and positional analysis of lysineFITC substituted peptides. (A) Exemplary SDS-PAGE analysis of affinity chromatography-purified dsSCD produced as Fc-free variants in CHO cells. dsSCD biotinylation was confirmed by gel shift upon equimolar addition (+) of streptavidin prior to the gel electrophoresis. (B) Systematic binding analysis of FITC-lysine (KFITC)-substituted NLVPMVATV peptides towards bead-immobilized HLA-A*02:01 dsSCD. Beads were pulsed with the indicated concentrations of FITCpeptides for 18 hours. Non-linear regression (one-site specific binding) of the FITC MFI against the peptide concentration and calculated KD values in µM are shown. For comparison, the NetMHCpan 4.1-based binding motif of naturally bound, eluted ligands (EL) of HLA-A*02:01 is shown above the lysine-substituted NLVPMVATV sequence (https://services.healthtech.dtu.dk/services/NetMHCpan-4.1/). HLA-A*02:01 %Rank EL prediction values are shown for the indicated peptides with K substitution and their classified binding levels as putative strong binders (SB, %Rank EL <0.5), weak binders (WB, %Rank EL <2) and non-binders (nonB, %Rank EL >2). The FITC conjugation is neglected by this prediction. [file Image_1.jpeg]

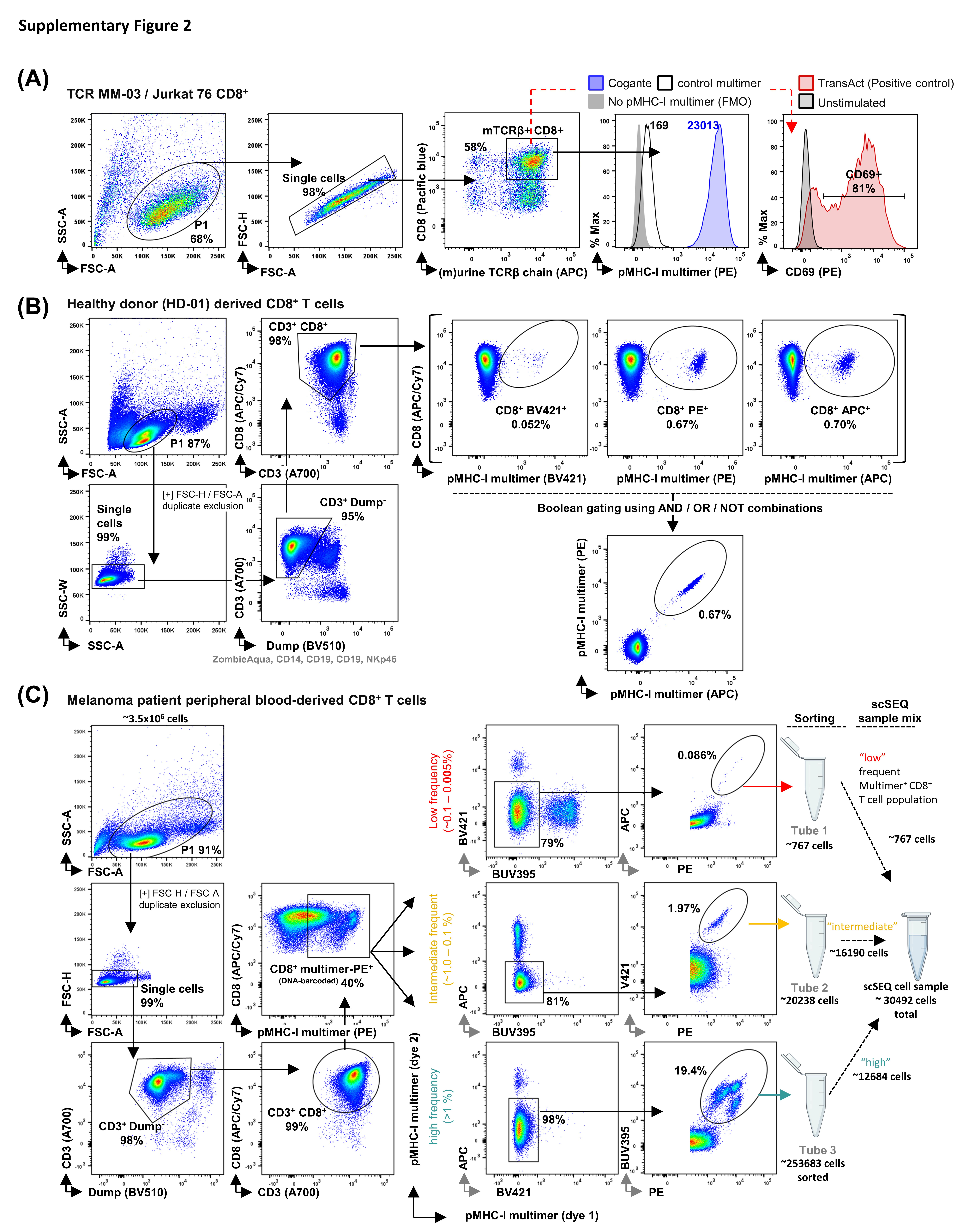

Supplement: Supplementary Figure 2 — Flow cytometry gating strategies. (A) Antigen-specific TCR validation of stably transfected Jurkat 76 CD8+ (J76CD8+) exemplary shown for the NY-ESO-1139-147/HLA-C*08:02-specific TCR MM-03 (see Figure 7 ). pMHC-I multimer binding (left histograms) or CD69 upregulation in the presence of stimulation (right histograms) is always shown for the TCR+/CD8+ J76CD8+ fraction. (B) Dual color-encoded pMHC-I multimer analysis of healthy donor (HD-01) and patient-derived CD8+ T cells is exemplary shown for one dual-color encoded pMHC-I multimer+ population of HD-01. HD-01 CD8+ T cells were stained with a pool of three dual-color pMHC-I multimer pairs, each pair associated with a different peptide and a unique dual color combination on the basis of streptavidin-conjugated fluorochromes APC, PE and BV421 used in this experiment for the pMHC-I multimer preparation. For analysis, single, living CD3+ CD8+ T cells were identified following the definition of positive events in each pMHC-I multimer channel as well as the generation of respective NOT gates thereof. To display a single dual color pMHC-I multimer population using Boolean gating, two positive gates (APC, PE) and here one NOT gate (BV421) are combined as a single AND gate (i.e., APC+ AND PE+ AND BV421–). Finally, the APC+ AND PE+ AND BV421– gate is combined as an OR gate with [APC– AND PE– AND BV421–] as shown for the HCMVpp65/A*02:02-specific CD8+ T cell population of HD-01. (C) Used gating strategy for cell sorting of dual color and DNA-barcoded (dCODE dextramers®) pMHC-I multimer+ CD8+ T cells of the melanoma patient for subsequent single-cell sequencing (scSEQ). Based on prior dual-color encoded pMHC-I multimer analysis of CD8+ T cells ex vivo from the patient, all identified pMHC-I multimer+ populations were pre-clustered for cell sorting depending on their frequency as low (< 0.1%), intermediate (0.1%–1.0%) or high (>1%) frequent and associated with the fluorochromes APC, BV421 or BUV395, respectively, to ensure that a [file Image_2.jpeg]

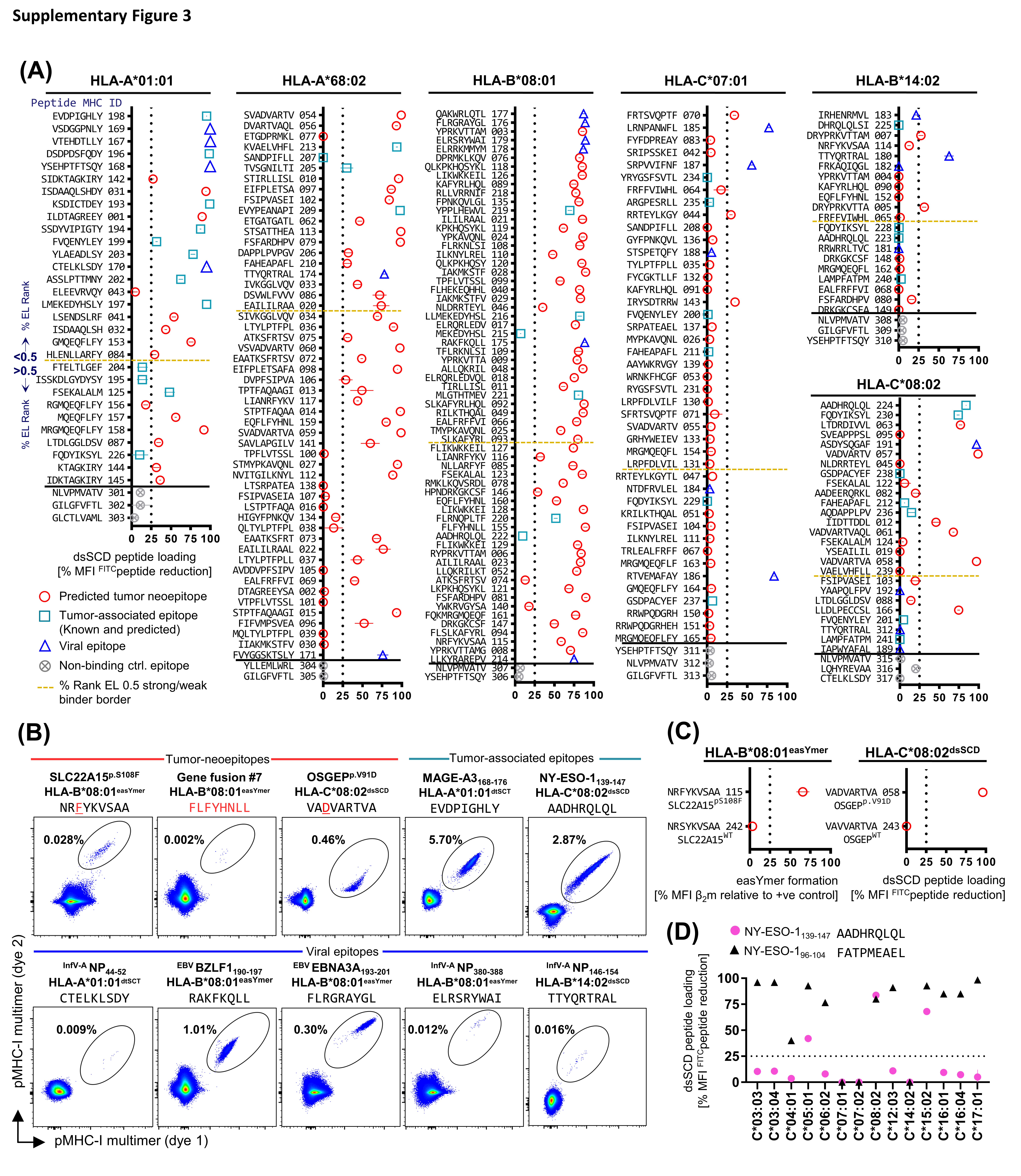

Supplement: Supplementary Figure 3 — dsSCD peptide binding validation of in silico predicted neoepitopes of a melanoma patient’s lymph node metastasis and identification of three neoepitope-specific and two TAA-specific CD8+ T cell populations in autologous peripheral blood. (A) HLA binding analysis of in silico predicted tumor neoepitopes and selected known and predicted tumor-associated non-mutated epitopes (TAA) using dsSCD-based peptide binding assays covering all six HLA-I allotypes of the patient. Shown is the MFI signal reduction ± SD in [%] relative to dsSCD-beads that have been loaded with FITCpeptide in the absence of a target peptide. Individual peptides are listed in top-down order according to their in silico binding prediction score (%Rank EL) for the respective HLA allotypes (also see Supplementary Table 3 ). Peptides are considered as HLA binders if they display a relative MFI reduction higher than 25% (dashed horizontal lines). (B) Labeling of the melanoma patient’s autologous peripheral CD8+ T cells ex vivo with dual-color encoded HLA-I multimers generated on the basis of dsSCD, dtSCT or commercial easYmer®. Representative dot plot of selected pMHC-I multimer+ populations that have been detected in three or more independent experiments are shown. (C) Comparative peptide-HLA binding analysis of predicted neoepitopes SLC22A15p.S108F and OSGEPp.V91D and their wild-type counterparts using an HLA-B*08:01 easYmer complex formation assay and HLA-C*08:02 dsSCD peptide binding assay, respectively. (D) Binding analysis of the NY-ESO-1139-147 (magenta) and NY-ESO-196-104 (black) peptide towards various HLA-C dsSCD allotypes. The experiments shown in (A, C, D) were conducted in technical triplicates. SD, Standard deviation of the mean. [file Image_3.jpeg]
